# GSH as an A‐A Type Allosteric Activator of PKM2: Modulating Cancer Cell Homeostasis and Ferroptosis Susceptibility

**DOI:** 10.1002/advs.202519368

**Published:** 2025-11-10

**Authors:** Tsan‐Jan Chen, Chi‐Jen Lo, Meng‐Jen Wu, Wei Yang Sit, Hsin‐Yu Hsu, Yi‐Cheng Huang, Chien‐Hung Lu, Yu‐Lun Chen, Wei‐Kai Fang, Shan‐Min Yang, Pei‐Lien Chen, Tokuko Haraguchi, Yasushi Hiraoka, Chun‐Yu Lin, Mei‐Ling Cheng, Muh‐Hwa Yang, Hsing‐Jien Kung, Wen‐Ching Wang

**Affiliations:** ^1^ Institute of Molecular and Cellular Biology and Department of Life Science National Tsing Hua University Hsinchu 300044 Taiwan; ^2^ Graduate School of Science Osaka University Toyonaka 560‐0043 Japan; ^3^ Metabolomics Core Laboratory Healthy Aging Research Center Chang Gung University Taoyuan 333323 Taiwan; ^4^ Clinical Metabolomics Core Laboratory Chang Gung Memorial Hospital Taoyuan 333423 Taiwan; ^5^ Institute of Bioinformatics and Systems Biology National Yang Ming Chiao Tung University Hsinchu 300093 Taiwan; ^6^ Graduate School of Frontier Biosciences Osaka University Suita 565–0871 Japan; ^7^ Center for Intelligent Drug Systems and Smart Bio‐devices National Yang Ming Chiao Tung University Hsinchu 300093 Taiwan; ^8^ Cancer and Immunology Research Center National Yang Ming Chiao Tung University Taipei 112304 Taiwan; ^9^ School of Dentistry Kaohsiung Medical University Kaohsiung 807378 Taiwan; ^10^ Department of Biomedical Sciences College of Medicine Chang Gung University Taoyuan 333323 Taiwan; ^11^ Institute of Clinical Medicine National Yang Ming Chiao Tung University Taipei 112304 Taiwan; ^12^ Ph.D. Program for Cancer Biology and Drug Discovery, College of Medical Science and Technology Taipei Medical University Taipei 110301 Taiwan; ^13^ Department of Biochemistry and Molecular Medicine Comprehensive Cancer Center University of California Davis, Sacramento CA 95817 USA

**Keywords:** cancer metabolism, ferroptosis, glutathione (GSH), lipid metabolism, pyruvate kinase M2 (PKM2)

## Abstract

This study identifies glutathione (GSH) as an endogenous A‐A type allosteric activator of pyruvate kinase M2 (PKM2), stabilizing it in its active tetrameric form through binding at the A‐A interface. This PKM2‐GSH interaction links GSH metabolism to ferroptosis regulation. Transcriptomic analyses across cancers demonstrate strong correlations between GSH, SLC7A11, PKM2, glycolysis, and ferroptosis pathways. By depleting GSH and activating PKM2, ferroptosis is enhanced in PKM2‐dependent cancer models. This approach leads to significant changes in central carbon and lipid metabolism, disrupts mitochondrial function, and drives ferroptotic cell death. The combined treatment markedly suppresses tumor growth in a xenograft model. Elevated PKM2 and SLC7A11 expression levels correlate with poorer survival outcomes, indicating their potential as biomarkers for ferroptosis‐based therapy. The findings demonstrate a dual role for GSH in cellular homeostasis and identify the PKM2‐GSH‐SLC7A11 axis as a therapeutic target for aggressive cancers.

## Graphical abstract

Glutathione (GSH) defines oxidative balance, but can it directly tune central metabolism? The research reveals that GSH binds and potently activates pyruvate kniase M2 (PKM2), rewiring glycolysis and dictating ferroptosis sensitivity. Combining GSH depletion with PKM2 activation triggers mitochondrial damage and lipid peroxidation, profoundly suppressing tumor growth in vivo. This unexpected vulnerability presents a promising new strategy for cancer therapy.

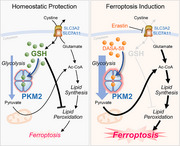

## 1 Introduction

Cancer metabolism, a hallmark of cancer biology, is marked by significant changes in cellular energy production and biosynthetic pathways that accommodate the demands of rapid cell proliferation and survival.^[^ ^1^, ^2^ ^]^ Among the most studied metabolic adaptations in cancer is the Warburg effect, where cancer cells preferentially utilize glycolysis for ATP production even in the presence of oxygen, resulting in the production of lactate rather than fully oxidizing glucose through the mitochondrial tricarboxylic acid (TCA) cycle.^[^ ^3^, ^4^ ^]^ This reliance on glycolysis, even under normoxic conditions, allows cancer cells to channel glycolytic intermediates into anabolic pathways, producing nucleotides, amino acids, and lipids required for biomass expansion.^[^ ^5^ ^]^ This metabolic reprogramming is governed by a network of enzymes and regulatory pathways, with the pyruvate kinase M2 (PKM2) enzyme being a critical modulator.^[^ ^6^, ^7^, ^8^ ^]^

PKM2 is an isoform of pyruvate kinase, an enzyme responsible for the final step in glycolysis, converting phosphoenolpyruvate (PEP) to pyruvate and generating ATP. PKM2 and PKM1 are alternative splice variants of the *PKM* gene. Unlike its isoform PKM1, which is constitutively active, PKM2 is subject to complex regulation, including allosteric modulation, post‐translational modifications, and interactions with various signaling molecules.^[^ ^9^ ^]^ PKM2 can adopt multiple oligomeric forms, with the R‐state tetrameric configuration being the most active.^[^ ^10^, ^11^ ^]^ The enzyme's activity is regulated by allosteric activators such as fructose 1,6‐bisphosphate (FBP), which promotes the formation of the active R‐state tetramer, and by inhibitors or somatic mutations that induce conformational changes leading to a less active state.^[^ ^9^, ^10^, ^11^ ^]^ In many cancers, PKM2 often exists in its dimeric or monomeric forms, which have lower catalytic activity, allowing the accumulation of upstream glycolytic intermediates that feed into anabolic pathways supporting cell proliferation and survival. Notably, PKM2 can translocate to the nucleus, where it functions as a co‐activator of transcription factors like hypoxia‐inducible factor 1‐alpha, driving the expression of genes essential for cell growth, survival, and angiogenesis.^[^ ^12^, ^13^, ^14^, ^15^, ^16^ ^]^

Beyond its role in facilitating rapid cell division, PKM2 also plays a vital role in managing cellular redox balance and oxidative stress.^[^ ^17^ ^]^ Cancer cells often experience elevated oxidative stress due to their high metabolic rates and mitochondrial dysfunction, which generate reactive oxygen species (ROS) that, if not properly managed, can lead to cellular damage or death.^[^ ^18^, ^19^ ^]^ PKM2 helps modulate this oxidative stress through metabolic adaptations that favor redox homeostasis. Under oxidative conditions, PKM2 is subject to redox‐sensitive modifications, such as the oxidation of cysteine residues, which leads to a shift in glucose metabolism toward the pentose phosphate pathway (PPP), an alternative pathway to glycolysis that generates reduced nicotinamide adenine dinucleotide phosphate (NADPH).^[^ ^17^ ^]^ NADPH is a crucial reducing agent that enables the regeneration of glutathione (GSH), one of the primary antioxidants within cells.^[^ ^20^ ^]^ Through these actions, PKM2 helps cancer cells maintain redox equilibrium, enabling them to survive and proliferate in the face of heightened oxidative stress.^[^ ^21^ ^]^

In recent years, ferroptosis has emerged as a distinct form of programmed cell death characterized by iron‐dependent lipid peroxidation and ROS accumulation.^[^ ^22^, ^23^, ^24^ ^]^ Unlike apoptosis and necroptosis, which are other forms of regulated cell death, ferroptosis involves the failure of cellular antioxidant systems, leading to an accumulation of lethal lipid peroxides, particularly within cell membranes.^[^ ^25^, ^26^ ^]^ A central player in preventing ferroptosis is the cystine/glutamate antiporter xCT, a heterodimeric transporter composed of SLC7A11 and SLC3A2, which imports cystine into the cell in exchange for glutamate.^[^ ^27^, ^28^ ^]^ Cystine is essential for synthesizing GSH, which, along with glutathione peroxidase 4 (GPX4), neutralizes lipid peroxides, thus preventing ferroptotic cell death.^[^ ^29^, ^30^ ^]^ Targeting xCT and other components of the GSH‐GPX4 pathway represents a promising therapeutic strategy, especially in cancers that depend heavily on antioxidant defenses to counteract high ROS levels.^[^ ^31^, ^32^ ^]^

Recent studies have suggested that PKM2 regulates ROS levels and oxidative stress. Oxidation of cysteine 358 in PKM2 has been shown to reduce its activity, redirecting glucose metabolism toward the PPP.^[^ ^17^ ^]^ This metabolic shift is crucial, as it increases the production of NADPH, a key reducing agent that supports the regeneration of GSH, enhancing the cell's antioxidant defenses. Additionally, excessive ROS generated under hypoxic conditions can further suppress PKM2 activity by destabilizing its tetrameric form through sulfhydration at cysteine 326, which limits glycolytic flux and redirects glucose toward antioxidant pathways.^[^ ^33^ ^]^ Given this role in managing redox balance, PKM2 could play a significant role in modulating ferroptosis, although the exact mechanisms remain incompletely understood.

This study identifies an unexpected role for GSH, a central antioxidant in cellular defense, as an allosteric activator of PKM2. Crystallographic structural analyses demonstrate that GSH stabilizes PKM2 in its active tetrameric form, enhancing glycolytic flux to support energy production while managing oxidative stress. This dual function of GSH suggests a complex role in cancer cell metabolism beyond its traditional antioxidant capacity. Data mining analysis reveals strong correlations between PKM2, SLC7A11, glycolysis, and ferroptosis pathways, suggesting that cancers with high abundance of PKM2 and elevated SLC7A11 expression exploit this PKM2‐GSH interplay to protect against ferroptosis while maintaining pyruvate production for energy and lipid synthesis. Our findings show that depleting GSH via SLC7A11 inhibition and directly activating PKM2 disrupts this protective mechanism, destabilizing redox balance and inducing ferroptotic cell death. These findings highlight a dual role of GSH in cancer metabolism and offer a promising therapeutic strategy targeting PKM2 and SLC7A11 in cancer cells highly dependent on redox adaptation.

## 2 Results

### 2.1 Identification of GSH as an Allosteric Activator of PKM2

Metabolic enzymes are often finely regulated by metabolites through allosteric interactions, allowing cells to adjust metabolic fluxes in response to changing conditions. PKM2, a key enzyme in glycolysis, is known to be allosterically modulated by various metabolites, including amino acids, nucleotides, and other small molecules integral to central metabolic pathways.^[^ ^34^ ^]^ For instance, FBP is a well‐established allosteric activator of PKM2, promoting a conformational shift from a less active tetramer (T‐state) to a highly active form (R‐state).^[^ ^10^ ^]^ Serine acts as an allosteric activator, enhancing PKM2 activity, whereas phenylalanine functions as an allosteric repressor.^[^ ^35^ ^]^ Succinyl‐5‐aminoimidazole‐4‐carboxamide‐1‐ribose‐5′‐phosphate (SAICAR), a nucleotide metabolite, has been shown to influence PKM2 activity allosterically.^[^ ^36^ ^]^

**Figure 1:**
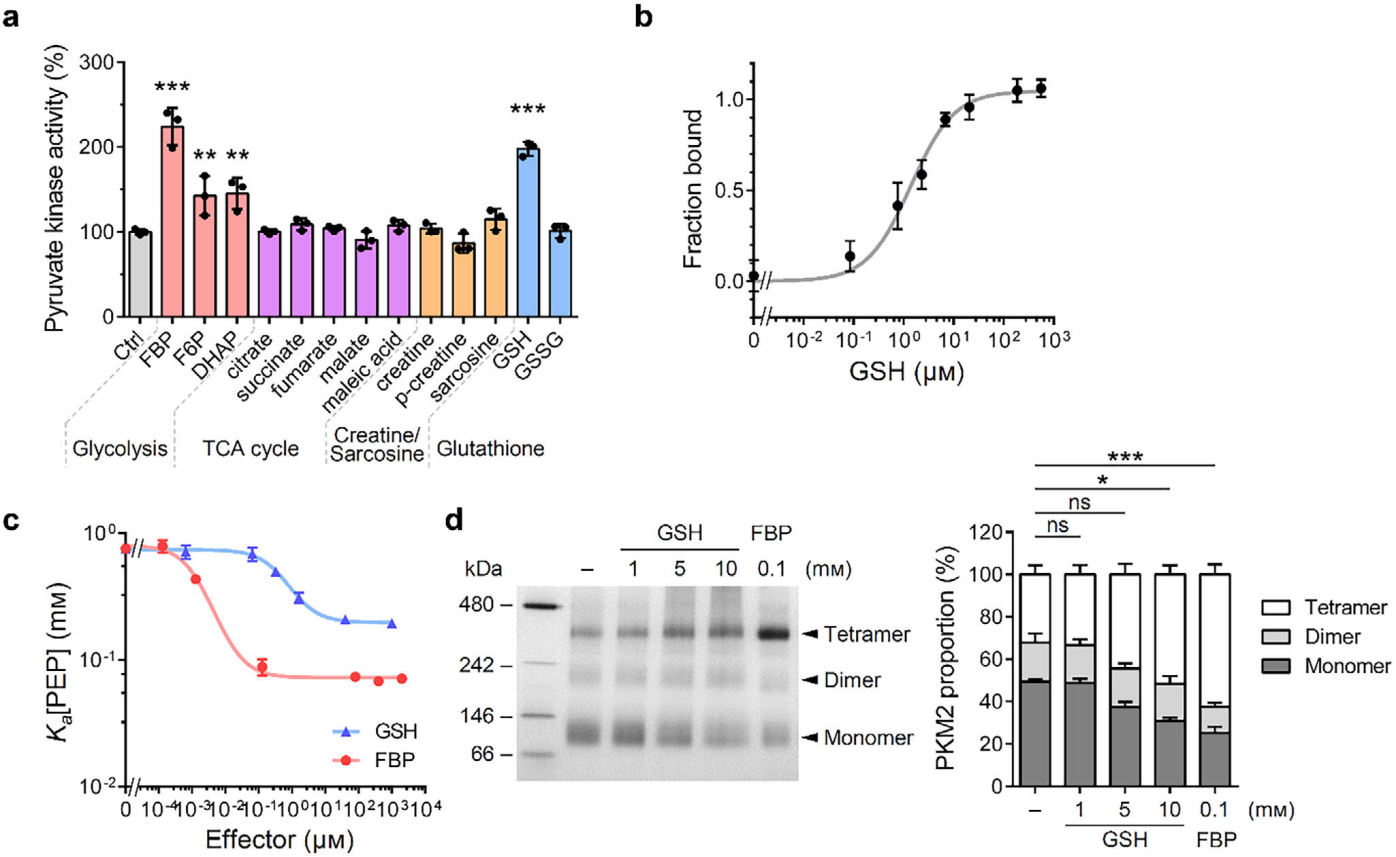
Allosteric activation of PKM2 by GSH. a) Metabolite screening using a pyruvate kinase activity assay. Recombinant PKM2 was incubated with 10 µм FBP, 100 µм GSH, and GSSG, or 1 mм of the other indicated metabolites prior to activity assay (*n* = 3). Ctrl, control; p‐creatine, phosphocreatine. Data significance is compared to the Ctrl group. b) MST assay conducted with EGFP‐tagged PKM2 to measure the binding affinity (*n* = 3). The 1.0 for the y‐axis indicates 100% ligand binding. The *K_d_* for GSH was calculated using the “One site‐specific binding” model and recorded as 1.38 ± 0.20 µм. c) Analysis of coupling constants (*Q_ax_*). The substrate affinity for PEP (*K_a_* [PEP]) was evaluated across various concentrations of the effectors, and the *Q_ax_* Values of FBP and GSH were calculated (*n* = 3). *Q_ax_* (FBP) = 11.0 ± 2.0. *Q_ax_* (GSH) = 3.7 ± 0.4. d) Blue native PAGE analysis revealed the oligomeric states of recombinant PKM2 incubated with effectors (*n* = 3). The gel was stained with Coomassie Brilliant Blue (left panel). The quantification result is shown in the right panel. Data significance is compared to the Ctrl group. Data are shown as mean ± SD for (a)‒(d). One‐way ANOVA with Bonferroni's multiple comparison test for (a). χ‐square test using the mean values as compared to the Ctrl group for (d). ^*^ *p* < 0.05, ^**^ *p* < 0.01, ^***^ *p* < 0.001, ns: *p* > 0.05.

Given PKM2's central role at the crossroads of glucose metabolism and its regulation by various metabolites, we hypothesized that additional metabolites within crucial metabolic pathways might allosterically modulate PKM2 activity. As PKM2 intercepts glucose metabolism and the metabolic network, we sought to investigate whether metabolites from key pathways, such as glycolysis, TCA cycle, creatine/sarcosine metabolism, and the glutathione pathway, could regulate PKM2's pyruvate kinase activity. A metabolite screening was conducted, focusing on central carbon metabolism pathways, utilizing an in vitro pyruvate kinase activity assay. Our results confirmed that FBP significantly increased PKM2 enzymatic activity by approximately twofold (**Figure** **1**a). Interestingly, glycolytic intermediates like fructose 6‐phosphate (F6P) and dihydroxyacetone phosphate (DHAP) also mildly enhanced PKM2 activity. Notably, our findings revealed that reduced GSH substantially upregulated PKM2 activity compared to its oxidized form, GSSG.

The activation of PKM2 by FBP and GSH occurred with half‐maximal effective concentrations (AC_50_) of 0.10 ±  0.02 and 98.09 ±  17.95 µм, respectively. Importantly, GSH did not affect lactate dehydrogenase (LDH) activity in the coupled reaction system (Figure S1a, Supporting Information). We also ruled out the possibility that GSH activates PKM2 through the reduction of cysteine residue C358, as the PKM2 C358A mutant remained responsive to GSH activation (Figure S1b, Supporting Information).^[^ ^17^ ^]^ Similarly to FBP, GSH did not influence the activity of PKM1, an isoform that is not allosterically regulated (Figure S1c, Supporting Information). While GSH allosterically activates only PKM2, both PKM1 and PKM2 isoforms can influence cellular GSH levels by directing glycolytic flux into or away from the PPP.

To assess whether a direct interaction occurred between PKM2 and GSH, we performed a microscale thermophoresis (MST) assay, which revealed a dissociation constant (*K_d_*) of 1.38 ± 0.20 µм between PKM2 and GSH (Figure 1b). The cytosolic concentration of GSH typically ranges from 1–10 mм, constituting 70%–90% of the total intracellular pool, whereas nuclear GSH levels are significantly lower at 0.1–1 mм.^[^ ^37^ ^]^ These compartment‐specific concentrations suggest that GSH‐mediated PKM2 activation may primarily occur in the cytoplasm, where GSH levels are sufficient to activate PKM2 efficiently under physiological conditions.

Given the biochemical and kinetic similarities between the effects of GSH and FBP, we proposed that GSH functions as an allosteric activator. We conducted a linked‐function analysis to evaluate the allosteric characteristics of GSH.^[^ ^38^ ^]^ The association constant (*K_a_*) of PEP was measured at various concentrations of FBP and GSH (Figure 1c). The *K_a_* values decreased with increasing concentrations of FBP and GSH, indicating enhanced affinity of PKM2 toward its substrate. The coupling constants, calculated as the ratio between the initial and final *K_a_* values, were 11.0 ± 2.0 for FBP and 3.7 ± 0.4 for GSH, demonstrating a positive allosteric effect on PKM2 activity.

Since FBP is known to stabilize PKM2 in its tetrameric form, leading to increased enzymatic activity, we investigated whether GSH similarly affects PKM2's oligomeric state. Blue native PAGE analysis using purified recombinant PKM2 incubated with GSH or FBP showed that GSH increased the proportion of tetrameric PKM2 in a dose‐dependent manner, akin to the effect observed with FBP (Figure 1d). These findings support the conclusion that GSH is an allosteric activator of PKM2.

### 2.2 GSH is an Endogenous A‐A Type Allosteric Activator of PKM2, Sharing a Common Binding Mechanism with DASA‐58 and TEPP‐46

**Figure 2:**
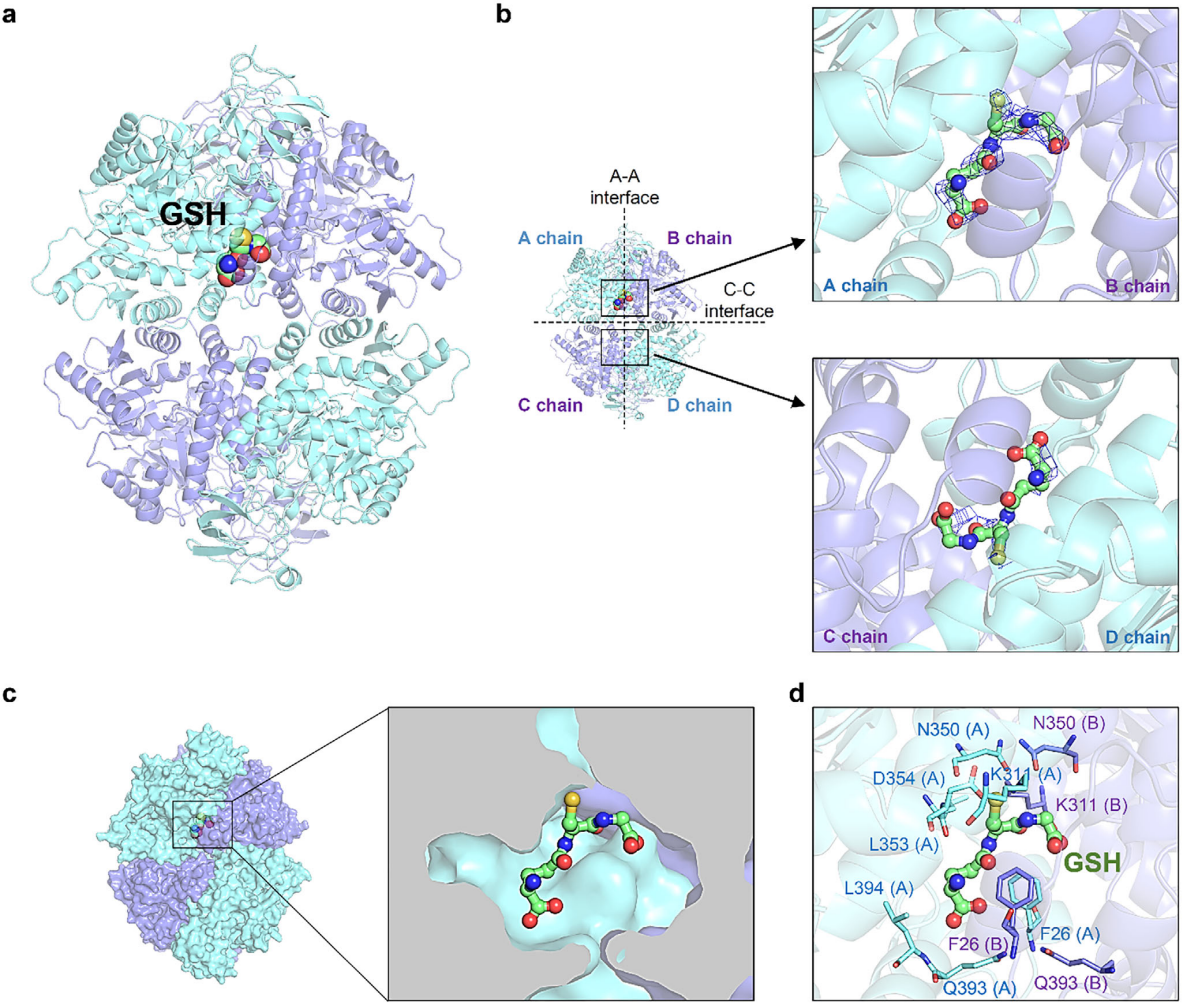
Crystal structure of the PKM2‐GSH complex. a) Crystal structure of the PKM2‐GSH complex (PDB: 9IQQ, this study), with PKM2 depicted in cyan and purple cartoon representations, and GSH shown in a sphere model. b) The tetrameric organization of PKM2, highlighting the A‐A and C‐C oligomerization interfaces along with the A, B, C, and D chains of the complex (left panel). The zoomed‐in views of the GSH binding site between chains A and B (upper panel) and at the symmetric pocket between chains C and D (lower panel) display the GSH molecule superimposed for comparison. The 2*F_o_*  −  *F_c_* electron density maps contoured at 1.1 σ are shown in blue mesh, with GSH represented in a stick‐and‐ball model. c) Surface view of the PKM2 tetramer (left panel) and a clipped view of the GSH binding site (right panel) showing the pocket where GSH binds. d) Close‐up of the residues involved in interactions with GSH, shown in stick representation. Chain identifiers of the residues are indicated in parentheses.

To investigate the atomic‐level interaction between PKM2 and GSH, we determined the crystal structure of PKM2 in complex with GSH at a resolution of 2.7 Å (**Figure** **2**a). The structure belongs to the P2_1_ space group, with some missing residues in the N‐terminal tail and B domain due to local flexibility. Over 95% of the residues exhibit preferred φ and ψ angles, ensuring the reliability of this model (PDB: 9IQQ). The overall structure aligns with the high‐activity R‐state conformation of PKM2, characteristic of its enzymatically active form. Structural statistics are summarized in Table S1 (Supporting Information).

In the electron density map, a distinct density corresponding to GSH was identified at the A‐A dimer interface between chains A and B (Figure 2b). Further analysis of the 2*F_o_*  −  *F_c_* and omit electron density maps confirmed the presence of bound GSH at this site (Figure 2b, upper panel; Figure S2, Supporting Information). The symmetric pocket between C and D chains also showed fragmented electron density signals, suggesting lower occupancy of GSH at this site (Figure 2b, lower panel). The surface view reveals that GSH binds in a pocket at the center of the dimerization (A‐A) interface (Figure 2c). Detailed structural analysis using PDBePISA identified key residues from chains A and B that interact with GSH through hydrogen bonds (Figure 2d; Table S2, Supporting Information). N350 and K311 from both A and B chains form hydrogen bonds with the sulfur and oxygen atoms in the cysteine moiety of GSH, while D354 in chain A also contributes a hydrogen bond with GSH's sulfur atom. The phenyl side chains of F26 residues from chains A and B pack against the GSH backbone, further stabilizing its binding.

**Figure 3:**
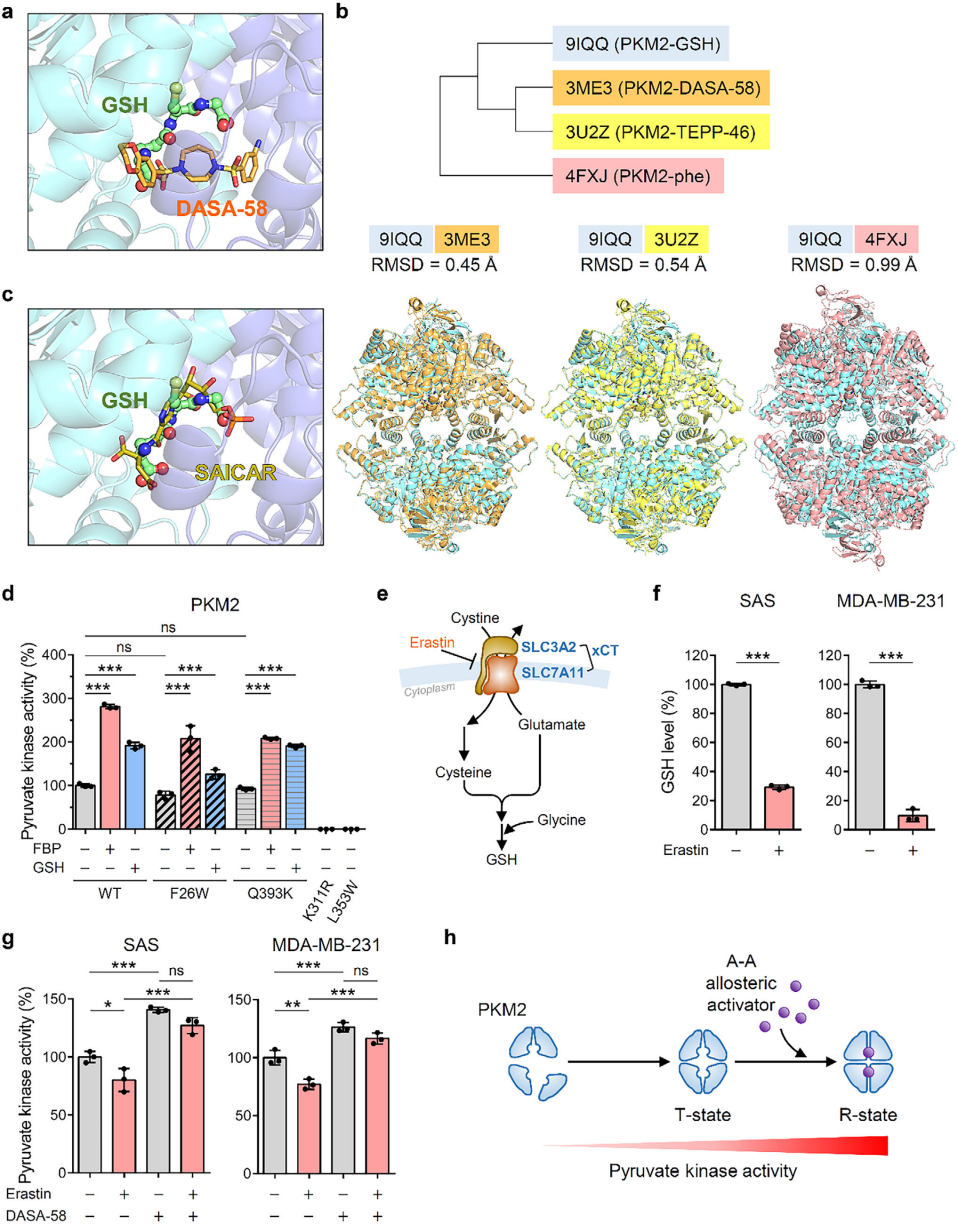
Shared binding pocket and conformational changes in PKM2 induced by GSH, DASA‐58, and TEPP‐46. a) Structural superimposition of GSH (shown as green stick‐and‐ball model in this study) and DASA‐58 (shown as orange sticks; PDB: 3ME3) within the PKM2 allosteric activator binding pocket, illustrating the overlapping binding sites of these two activators. b) Phylogenetic tree analysis of PKM2 and related structures based on RMSD values (upper panel). Each structure is compared to the GSH‐bound PKM2 structure (this study, PDB: 9IQQ). The lower panel shows superimpositions of the GSH‐bound PKM2 structure with other PKM2 states, including the PKM2‐DASA‐58 complex (PDB: 3ME3), PKM2‐TEPP‐46 complex (PDB: 3U2Z), and T‐state PKM2 (PDB: 4FXJ), highlighting their structural similarities and differences. c) Computational docking of SAICAR into the A‐A allosteric pocket of PKM2, overlaid with the crystallographically determined GSH position (this study). GSH is shown as green sticks and balls, and SAICAR as ochre sticks. d) Pyruvate kinase activity of recombinant His‐tagged PKM2 variants (WT, F26W, Q393K, K311R, and L353W) assayed with or without 100 µм FBP or 100 µм GSH (*n* = 3). e) Schematic diagram showing erastin as an inhibitor of SLC7A11/xCT, a cystine/glutamate antiporter crucial for GSH biosynthesis. f) Intracellular GSH level normalized to total protein level. SAS and MDA‐MB‐231 cells were treated with 2 and 10 µм erastin for 24 h, respectively, followed by GSH quantification (*n* = 3). g) Pyruvate kinase activity normalized to PKM2 levels. Cells were treated with various combinations of effectors (SAS: 5 µм erastin and/or 10 µм DASA‐58 for 6 h; MDA‐MB‐231: 10 µм erastin and/or 20 µм DASA‐58 for 24 h), and total pyruvate kinase activity was measured (*n* = 3). h) Schematic representation of PKM2 conformational changes triggered by allosteric activation. The transition from a less active conformation to a high‐activity tetrameric R‐state is induced by the binding of A‐A type activators such as GSH or DASA‐58. Data are shown as mean ± SD for (d), (f), and (g). One‐way ANOVA with Tukey's multiple comparison tests for (d) and (g). Two‐tailed unpaired Student's *t*‐test for (f). ^*^ *p* < 0.05, ^**^ *p* < 0.01, ^***^ *p* < 0.001, ns: *p* > 0.05.

We conducted a structural comparative analysis of the PKM2‐GSH complex with known PKM2‐liganded structures. Interestingly, when the PKM2‐GSH structure was superimposed with previously solved PKM2 structures bound to the small‐molecule activators DASA‐58 (PDB: 3ME3) and TEPP‐46 (PDB: 3U2Z), we observed an overlap in their binding sites at the A‐A interface (**Figure** **3**a; Figure S3a, Supporting Information).^[^ ^39^ ^]^ Root mean square deviation (RMSD) calculations for C_α_ atoms revealed that the PKM2‐GSH complex is structurally closest to the PKM2‐DASA‐58 complex (RMSD = 0.45 Å) and most distant from the T‐state PKM2 structure (RMSD = 0.99 Å) (Figure 3b). These findings support the notion that GSH, DASA‐58, and TEPP‐46 induce similar conformational changes in PKM2 to promote the formation of the active tetramer.

Since SAICAR, a PKM2 allosteric activator, binds near Q393 with a *K_d_* of 300 µм,^[^ ^40^ ^]^ we asked whether SAICAR could compete with GSH for the same allosteric site on PKM2. Computational docking of SAICAR into our PKM2‐GSH structure revealed that both ligands occupy overlapping positions at the same A‐A interface pocket (Figure 3c). In this model, SAICAR and GSH each hydrogen bond to N350 and K311 on both subunits. Notably, only SAICAR, but not GSH, hydrogen bonds with Q393 (chain A), while F26 from each subunit makes van der Waals contacts with both ligands (Figure S3b, Supporting Information).

We conducted an in vitro displacement assay using a PKM2 pull‐down assay, followed by GSH detection through luminescence. Recombinant His‐tagged PKM2 was preloaded with GSH, washed, and subsequently challenged with either SAICAR (5 mм) or FBP (5 mм) (Figure S3c, Supporting Information). SAICAR, but not FBP, significantly displaced bound GSH from PKM2 (Figure S3d, Supporting Information), indicating shared occupancy of the same allosteric pocket.

We next generated four A‐A interface mutants to probe their roles in effector binding: K311R, L353W, F26W, and Q393K. K311R, and L353W essentially abolished PKM2 basal activity, confirming the functional importance of this region (Figure 3d). By contrast, F26W retained full responsiveness to FBP but showed only partial activation to GSH, whereas Q393K exhibited a behavior comparable to that of wild type (WT) with both effectors (Figure 3d). Although Q393K disrupts SAICAR binding,^[^ ^36^ ^]^ it remains fully responsive to GSH. Structurally, F26 packs directly against GSH, so its mutation to tryptophan greatly hinders binding, while Q393 sits at the rim and contributes little to GSH binding (Figure S3b, Supporting Information). Together, these results suggest F26 as the critical residue for GSH's allosteric activation of PKM2.

To assess the cellular relevance of GSH binding on PKM2 activity, we investigated whether GSH depletion affects pyruvate kinase activity in SAS and MDA‐MB‐231 cells, which exhibit high PKM2 dependency according to the DepMap database analysis (Figure S3e, Supporting Information). Erastin, an inhibitor of SLC7A11, effectively depleted intracellular GSH levels (Figure 3e,f). This GSH depletion significantly reduced pyruvate kinase activity, supporting the reliance of PKM2 on GSH for its activity (Figure 3g). We then examined whether DASA‐58, which does not contain the thiol moiety but acts as a PKM2 activator, could restore PKM2 activity under GSH‐depleted conditions. Notably, DASA‐58 treatment significantly rescued pyruvate kinase activity in GSH‐depleted cells (Figure 3g). Western blotting analysis confirmed that PKM2 protein levels remained constant across all treatments (Figure S3f, Supporting Information), and although erastin alone elicited a slight upregulation of SLC7A11 in MDA‐MB‐231 and SAS cells, co‐treatment with DASA‐58 had no further effect on its expression.

Using a native PKM2 pull‐down assay followed by GSH detection, we showed that endogenous GSH is physically associated with PKM2 in cells, and that erastin treatment abolishes this association (Figure S3g, Supporting Information). To verify that the loss of PKM2 activity reflects GSH depletion, we rescued intracellular GSH in SAS and MDA‐MB‐231 cells using cell‐permeable glutathione ethyl ester (GSH‐EE). Remarkably, GSH‐EE fully restored pyruvate kinase activity in erastin‐treated cells (Figure S3h, Supporting Information). Together, these results suggest that GSH functions as an endogenous A‐A type allosteric activator of PKM2 and classify DASA‐58 and TEPP‐46 as pharmacological mimics that stabilize PKM2 in its active conformation through the same allosteric mechanism (Figure 3h). This shared binding and activation pathway presents a potential means of targeting PKM2 activity in cancer cells.

### 2.3 Correlation Analysis Reveals the Interplay between PKM2, GSH, SLC7A11, Glycolysis, and Ferroptosis

**Figure 4:**
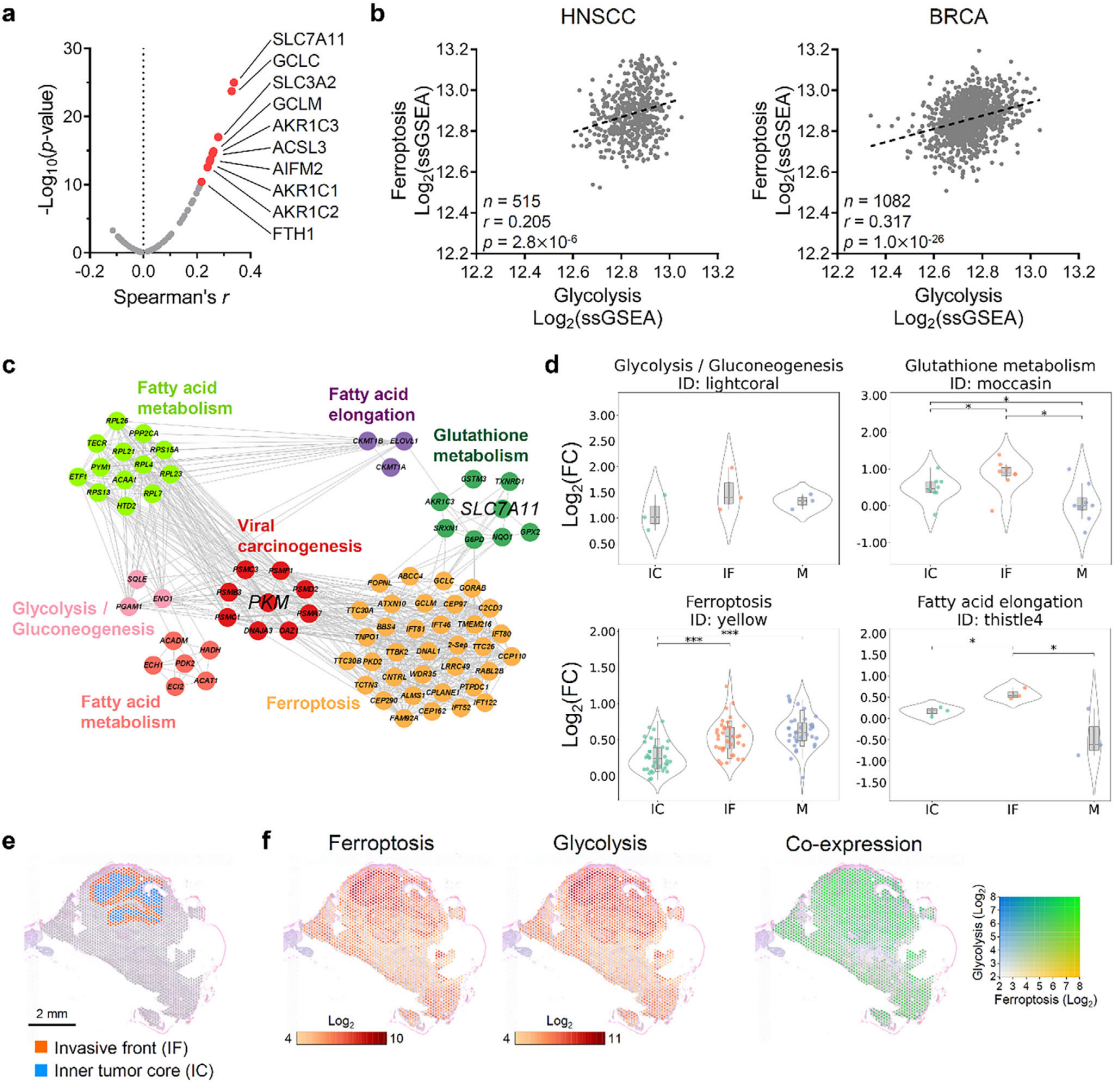
Correlation of *PKM*, *SLC7A11*, glycolysis, and ferroptosis pathways. a) The correlation between gene expression levels [Log_2_(TPM + 1)] and GSH abundance (Log_10_ scale) across 911 cell lines from the DepMap portal. The analysis focused on the WP_FERROPTOSIS gene set (MSigDB), with Spearman's correlation coefficient (*r*) and the ‐Log_10_(*p*‐value) indicated for each gene. The top 10 positively correlated genes are highlighted in red dots. b) Correlation analysis in HNSCC and BRCA cohorts. Scores for ferroptosis and glycolysis signatures in the TCGA Pan‐Cancer HNSCC and BRCA cohorts were calculated using the single‐sample gene set enrichment analysis (ssGSEA) module (GenePattern) with gene sets WP_FERROPTOSIS and HALLMARK_GLYCOLYSIS (MSigDB). The Log_2_‐transformed scores were plotted against the y and x axes. The Spearman's correlation coefficient (*r*) and the *p*‐values for each analysis are shown. c) MMI subnetworks centered on *SLC7A11* and *PKM*, highlighting enriched modules related to glutathione metabolism, ferroptosis, glycolysis/gluconeogenesis, fatty acid elongation/metabolism, and viral carcinogenesis. d) Violin plots of genes in the modules enriched in the pathways of glycolysis/gluconeogenesis, glutathione metabolism, ferroptosis, and fatty acid elongation. HNSCC samples derived from IC, IF, and M are compared. Each dot represents the Log_2_(tumor vs normal fold change) [Log_2_(FC)] value for a gene within the module with the corresponding enriched pathway. A boxplot in the center of each violin plot is used to show the minimum, first quartile, median, third quartile, and maximum. Wilcoxon signed‐rank test. ^*^ *p* < 0.05, ^**^ *p* < 0.01, ^***^ *p* < 0.001. e) Spatial transcriptomic analysis of an HNSCC tumor section using the Visium Spatial Gene Expression platform (10x Genomics). Orange dots indicate IF sites, and blue dots represent IC sites.^[^ ^43^ ^]^ f) Expression levels of ferroptosis signature (WP_FERROPTOSIS) and glycolysis signature (HALLMARK_GLYCOLYSIS) are shown. The co‐expression profile between ferroptosis and glycolysis signatures is shown as a heat map, where the green color indicates a high degree of co‐enrichment.

Given the critical role of PKM2 activity in cancer cell survival and its potential regulation by GSH, we hypothesized that cancer cells might exploit the PKM2‐GSH interaction to modulate ferroptosis, a form of iron‐dependent cell death associated with lipid peroxidation. GSH is essential for protecting cells against ferroptosis by maintaining redox homeostasis and neutralizing ROS.^[^ ^29^, ^30^ ^]^ To investigate this hypothesis, we used the DepMap cell line database to assess how genes related to glutathione metabolism and GSH levels across a wide range of cancer cell lines.^[^ ^40^ ^]^ Spearman's correlation analysis identified *SLC7A11*, which encodes the xCT cystine/glutamate antiporter, as the gene most significantly correlated with GSH levels (Figure S4a, Supporting Information). This strong correlation suggests the critical role of xCT in importing cystine, the precursor for GSH synthesis, thus maintaining intracellular GSH levels and contributing to ferroptosis resistance. We further examined the WP_FERROPTOSIS pathway (MSigDB).^[^ ^41^ ^]^ Within this pathway, *SLC7A11* again showed the highest correlation with GSH levels (**Figure** **4**a), reinforcing its central role in the regulation of ferroptosis through glutathione metabolism. This finding further positions xCT not only to support antioxidant defenses but may also influence glycolytic metabolism via the PKM2‐GSH axis.

To explore the broader connection between ferroptosis and cancer metabolism, we conducted a pan‐cancer analysis using the Cancer Genome Atlas (TCGA) database.^[^ ^42^ ^]^ This analysis revealed a consistent positive correlation between ferroptosis, ROS pathways, and glycolysis signatures across various cancer types, including head and neck squamous cell carcinoma (HNSCC) and breast cancer (BRCA) (Figure 4b; Figure S4b, Supporting Information).

It is also noted that there is a positive correlation between *SLC7A11* and *PKM* expression in HNSCC and BRCA cell lines (Figure S4c, Supporting Information). This co‐expression suggests a coordinated upregulation of PKM2 and SLC7A11 in cancer cells with high GSH levels.

### 2.4 Spatial Transcriptomic Analyses of HNSCC Tumor Regions Focused on PKM2 and SLC7A11

To investigate the metabolic heterogeneity across different tumor regions, we performed weighted gene co‐expression network analysis (WGCNA) on RNA‐seq data (GEO ID: GSE178537) from 65 samples in 21 HNSCC patients.^[^ ^43^ ^]^ This analysis exploited Gene Ontology (GO) biological processes and Kyoto Encyclopedia of Genes and Genomes (KEGG) pathways to identify co‐expression patterns associated with specific metabolic functions. The WGCNA identified multiple gene modules enriched in specific biological pathways, including oxidative phosphorylation, glycolysis, glutathione metabolism, and ferroptosis (Figure S5a, Supporting Information). These modules represent groups of genes with highly correlated expression profiles, suggesting coordinated regulation and potential functional relationships within these metabolic pathways.

To further explore the interactions among these metabolic modules, we constructed a module‐module interaction (MMI) network (Figure S5b, Supporting Information). This network visualization revealed significant interactions between modules centered on *PKM* and *SLC7A11* (Figure 4c). Analyzing gene expression across primary tumor regions, inner tumor core (IC), invasive front (IF), and metastasis (M), we observed distinct patterns of metabolic activity. Notably, the IF region exhibited marked upregulation of genes involved in key metabolic pathways (Figure S5c, Supporting Information). To quantitatively assess the enrichment of specific metabolic pathways in these regions, we generated violin plots illustrating the expression levels of genes linked to glycolysis, glutathione metabolism, fatty acid metabolism, ferroptosis, and related pathways (Figure 4d; Figure S5d, Supporting Information). These plots demonstrated significant enrichment of these pathways at the IF site compared to the IC and M regions. The elevated expression of glycolytic and antioxidant pathways at the IF suggests that cancer cells at the invasive front are metabolically reprogrammed to meet the energy demands of invasion and to counteract the oxidative stress associated with this process.

To validate our findings within a spatial context, we utilized a spatial transcriptomic dataset of an HNSCC tumor section (GEO ID: GSE181300), which provided high‐resolution spatial gene expression data with IF and IC regions (Figure 4e).^[^ ^43^ ^]^ Our spatial analysis revealed that signatures associated with ferroptosis (WP_FERROPTOSIS) and glycolysis (HALLMARK_GLYCOLYSIS) were significantly enriched at the IF (Figure 4f). Moreover, we observed that the mRNA expression levels of *PKM* and *SLC7A11* were co‐expressed with ferroptosis signatures at the IF (Figure S6a,b, Supporting Information). This co‐expression pattern indicates a potential synergistic role of *PKM* and *SLC7A11* in regulating metabolic pathways that confer survival advantages to cancer cells at the invasive front. Consistent with the ferroptosis signature, *GCLM* and *GCLC*, which are key enzymes responsible for GSH biosynthesis, were markedly enriched alongside PKM2 at the IF region relative to the IC region in the spatial transcriptomic analysis, suggesting enhanced GSH synthesis at the tumor periphery (Figure S6c, Supporting Information). We then performed dual immunohistochemistry (IHC) analysis on an independent cohort of 15 HNSCC resections, finding strong co‐localization of PKM2 and high GSH staining in 10 out of 12 IF regions but only one out of six IC regions, indicating a pronounced spatial association of these metabolic markers at the invasive front (Figure S6d, Supporting Information). To evaluate whether this peripheral PKM2‐GSH coupling extends to other tumor types, we further analyzed bulk RNA sequencing data from TCGA for two types of cancers: lung adenocarcinoma (LUAD) and lung squamous cell carcinoma (LUSC). In each cohort, we observed a significant positive correlation between PKM2 and the GSH‐biosynthetic genes *GCLM*/*GCLC* (Spearman's *r* = 0.18‐0.40, *p* < 1 × 10^−4^) (Figure S6e, Supporting Information). These findings together indicate that upregulation of PKM2 at the tumor edge co‐occurs with enhanced GSH synthesis, pointing to a conserved spatial PKM2‐GSH axis that may underlie aggressive growth and ferroptosis modulation.

To ask whether PKM2 level is directly correlated with ferroptosis vulnerability, we first surveyed PKM2 expression in various cancer types using the TCGA database. The result suggests that PKM2 is significantly upregulated in most cancer types, including BRCA, HNSCC, LUSC, and LUAD (Figure S7a, Supporting Information). We then examined the DepMap portal, comparing *PKM* (mRNA) expression to erastin sensitivity across HNSCC and BRCA cell lines (Figure S7b, Supporting Information). Our analysis showed that higher *PKM* levels in HNSCC and BRCA cell lines are associated with increased erastin sensitivity (indicated by reduced AUC values), suggesting the role of high PKM2 levels as a biomarker for ferroptosis vulnerability.

### 2.5 PKM2 Activation Sensitizes GSH‐Depleted Cancer Cells to Ferroptosis by Enhancing Oxidative Stress and Glycolytic Flux

**Figure 5:**
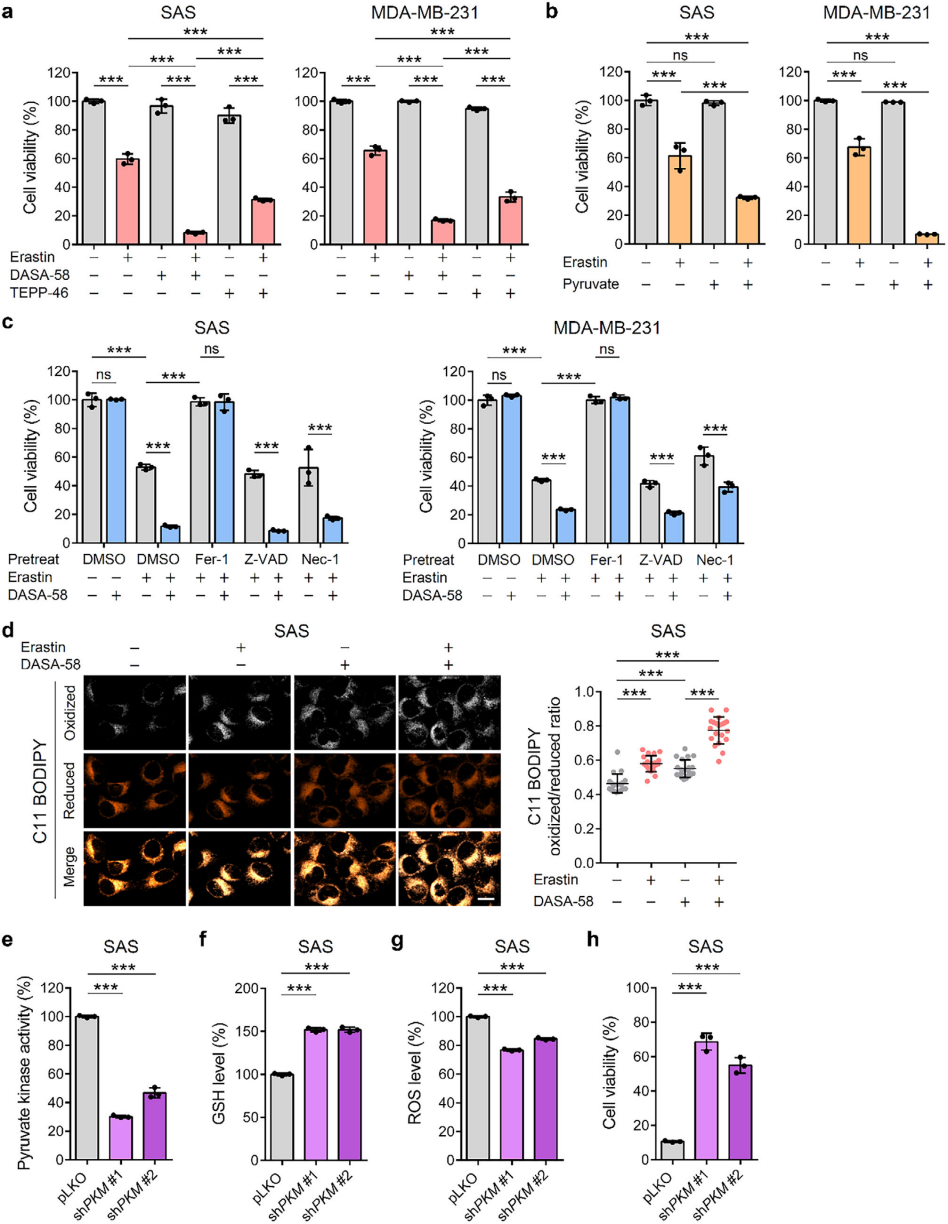
PKM2 activation enhances ferroptotic cell death in GSH‐depleted cells. a) Cell viability measured by the MTT assay. Cells were treated with different combinations of effectors for 24 h (SAS: 4 µм erastin, 20 µм DASA‐58, or 20 µм TEPP‐46; MDA‐MB‐231: 3 µм erastin, 20 µм DASA‐58, or 20 µм TEPP‐46; *n* = 3). b) Cell viability under treatment with erastin and pyruvate. Cells were treated with erastin and 5 mм pyruvate for 24 h (SAS: 4 µм erastin; MDA‐MB‐231: 3 µм erastin) and assessed using the MTT assay (*n* = 3). c) Rescue of cell viability by cell death inhibitors. SAS and MDA‐MB‐231 cells were pretreated for 1 h with dimethyl sulfoxide (DMSO), ferrostatin‐1 (Fer‐1, 5 µм), Z‐VAD(OMe)‐FMK (Z‐VAD, 20 µм), or necrostatin‐1 (Nec‐1, 20 µм). The medium was then replaced, and effector combinations (3 µм erastin and/or 10 µм DASA‐58 for SAS, 3 µм erastin and/or 20 µм DASA‐58 for MDA‐MB‐231) were added for another 24 h, followed by MTT assay (*n* = 3). d) C11 BODIPY staining for lipid peroxidation. SAS cells were treated with or without 2 µм erastin and/or 5 µм DASA‐58 for 24 h, followed by C11 BODIPY staining. Representative images showing oxidized (white) and reduced (red) C11 BODIPY signals, and merged images are shown (left panel). Scale bar = 20 µm. Quantification of oxidized/reduced C11 BODIPY ratio is shown (right panel, *n* = 20). e) Pyruvate kinase activity in control (pLKO) and PKM2‐knockdown (sh*PKM*) SAS cells (*n* = 3). f) Intracellular GSH levels in pLKO and sh*PKM* SAS cells (*n* = 3). g) Intracellular ROS levels in pLKO and sh*PKM* SAS cells quantified by 2′,7′‐dichlorodihydrofluorescein diacetate (H2DCFDA) fluorescence normalized to total protein (*n* = 3). h) Cell viability in pLKO and sh*PKM* SAS cells treated with 6 µм erastin for 24 h, measured by MTT assay (*n* = 3). Untreated cell viability was defined as 100% (data not shown). Data are shown as mean ± SD for (a)‒(h). One‐way ANOVA with Tukey's multiple comparison tests for (a),(b),(d)‒(h). Two‐way ANOVA with Tukey's multiple comparison test for (c). ^*^ *p* < 0.05, ^**^ *p* < 0.01, ^***^ *p* < 0.001, ns: *p* > 0.05.

Given that the PKM2‐GSH interaction is crucial for maintaining redox homeostasis and activating glycolysis, we explored the possibility of targeting the PKM2‐GSH‐SLC7A11 axis to regulate ferroptosis and influence cancer cell survival. To test this proposal, we examined the impact of PKM2 activation under GSH‐depleted conditions using SAS and MDA‐MB‐231 cell lines. These cell lines were chosen because they exhibit high levels of both SLC7A11 and PKM2 and have a strong dependency on PKM2 activity, as indicated by the DepMap database analysis (Figure S3e; Figure S8a, Supporting Information). We utilized pharmacological PKM2 activators, DASA‐58 and TEPP‐46, in our experiments. We observed that the combination of erastin (a GSH‐depleting agent) and DASA‐58 significantly suppressed cell viability more than either agent alone (**Figure** **5**a). A similar synergistic effect was noted with TEPP‐46 under GSH‐depleted conditions. The combination of erastin and DASA‐58 also led to a significant loss of viability in U87 glioblastoma and H1299 non‐small cell lung carcinoma cells (Figure S8b, Supporting Information). Comparable findings were also noted using another ferroptosis inducer, RSL3,^[^ ^44^ ^]^ indicating synergistic effects across various ferroptosis inducers and different cell types (Figure S8c, Supporting Information). Interestingly, supplementing cells with exogenous pyruvate and FBP also enhanced erastin's lethality (Figure 5b; Figure S8d, Supporting Information). These results suggest that enhanced glycolytic flux contributes to the induction of ferroptosis when antioxidant defenses are compromised.

To assess how PKM2 activation affects oxidative stress under GSH‐depleted conditions, we measured ROS levels following erastin treatment.^[^ ^45^ ^]^ Erastin‐induced GSH depletion elevated ROS levels, which were further increased by DASA‐58 co‐treatment (Figure S8e, Supporting Information). To determine the specific mode of cell death induced by the combination treatment, we pre‐treated cells with specific inhibitors: ferrostatin‐1 (a ferroptosis inhibitor), Z‐VAD(OMe)‐FMK (an apoptosis inhibitor), or necrostatin‐1 (a necroptosis inhibitor), followed by erastin and DASA‐58 treatment. Only ferrostatin‐1 rescued cell viability (Figure 5c), confirming that the cell death observed was primarily due to ferroptosis. Additionally, C11 BODIPY staining showed elevated lipid peroxidation, a hallmark of ferroptosis, upon erastin treatment, which was further enhanced by DASA‐58 co‐treatment (Figure 5d; Figure S8f, Supporting Information).

To further investigate the role of PKM2 in this ferroptotic process, we generated *PKM* knockdown cells in SAS and MDA‐MB‐231 cells. These knockdown cells showed reduced pyruvate kinase activity, elevated GSH levels, reduced ROS levels, and increased cell viability under erastin treatment compared to control cells (Figure 5e‒h; Figure S8g‒k, Supporting Information), suggesting that PKM2 downregulation provides a survival advantage under ferroptotic conditions by redirecting flux into the pentose phosphate pathway.

### 2.6 F26W Hinders GSH Binding and Uncouples PKM2 from Ferroptotic Regulation

**Figure 6:**
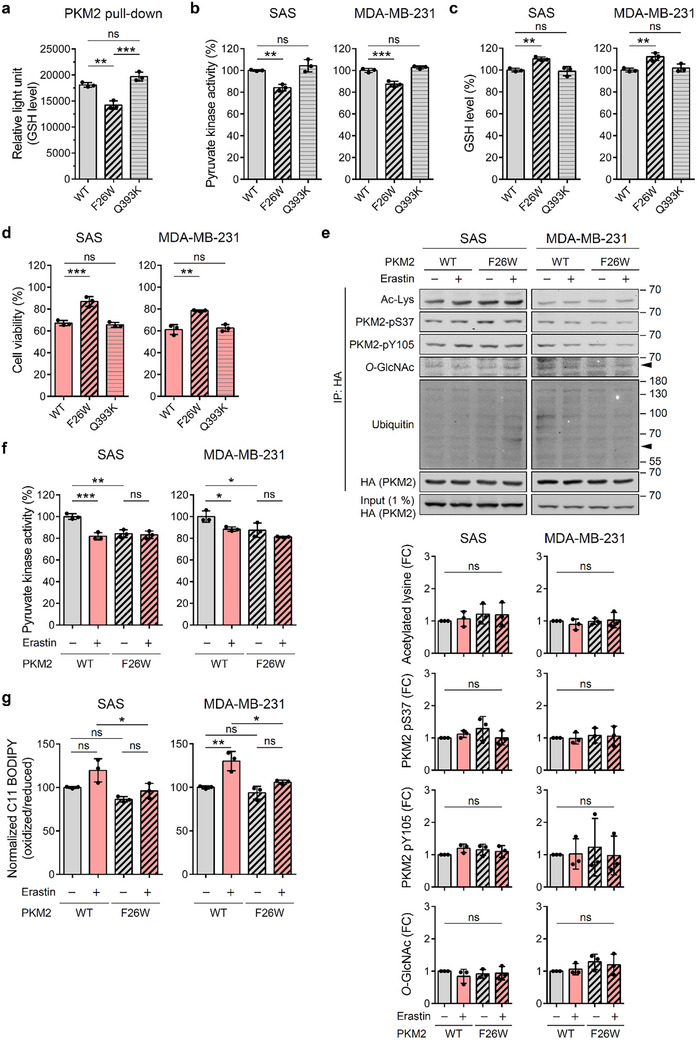
F26W reduces GSH binding and uncouples PKM2 from ferroptotic regulation. a) In vitro PKM2‐GSH pull‐down and quantification. His‐tagged PKM2 variants were incubated with GSH (2.5 mм), loaded onto the affinity resin for 30 min, washed, and the bound GSH was quantified by luminescence assay (*n* = 3). b) Pyruvate kinase activity in PKM2‐knockdown cells re‐expressed with HA‐tagged WT, F26W, or Q393K PKM2, normalized to total protein level (*n* = 3). c) Intracellular GSH levels in the same cell lines, measured by luminescence assay and normalized to total protein (*n* = 3). d) Cell viability of WT‐, F26W‐, or Q393K‐expressing PKM2‐knockdown cells following 24 h of treatment with erastin (4 µм for SAS; 6 µм for MDA‐MB‐231), measured by MTT assay (*n* = 3). Untreated controls are defined as 100%. e) PKM2 PTM profiling. After 24 h of treatment (with or without erastin), HA‐tagged WT or F26W PKM2 were immunoprecipitated, and the PTM levels (acetylated lysine, pS37, pY105, *O*‐GlcNAcylation, and ubiquitination) were analyzed by Western blotting. Representative blots are shown in the upper panel, and quantification of PTM levels (fold change relative to untreated WT) is presented in the lower panel (*n* = 3). Ubiquitination was not quantified due to weak signals. Ac‐Lys, acetylated lysine. f) Pyruvate kinase activity in PKM2‐knockdown cells re‐expressing WT or F26W PKM2, treated with or without erastin for 24 h (4 µм for SAS; 6 µм for MDA‐MB‐231), normalized to total protein level (*n* = 3). g) C11 BODIPY staining of lipid peroxidation in PKM2‐knockdown cells re‐expressing WT or F26W PKM2 after 24 h of erastin treatment (4 µм for SAS; 6 µм for MDA‐MB‐231). The normalized oxidized/reduced C11 BODIPY ratio is shown, with the untreated WT group set to 100%. Data are shown as mean ± SD for (a)‒(g). One‐way ANOVA with Tukey's multiple comparison tests for (a)‒(g). ^*^ *p* < 0.05, ^**^ *p* < 0.01, ^***^ *p* < 0.001, ns: *p* > 0.05.

To validate the F26W mutant as a GSH‐binding‐deficient PKM2 variant, we performed GSH pull‐down assays and confirmed that F26W exhibited markedly reduced binding compared with WT, whereas the Q393K mutant retained GSH interaction (**Figure** **6**a).

To examine the roles of F26 and Q393 in cellular regulation, we re‐expressed WT, F26W, or Q393K in PKM2‐depleted SAS and MDA‐MB‐231 lines (Figure S9a, Supporting Information). F26W‐expressing cells exhibited reduced pyruvate kinase activity, elevated GSH, and significantly greater resistance to erastin‐induced death compared to WT (Figure 6b‒d), consistent with the in vitro findings. By contrast, Q393K‐expressing cells were essentially indistinguishable from WT, maintaining full PKM2 activity, normal GSH levels, and erastin sensitivity (Figure 6b–d). Notably, simple overexpression of PKM2 in parental cells did not further sensitize them to erastin (Figure S9b, Supporting Information).

We next investigated whether erastin‐induced GSH depletion alters PKM2 post‐translational modifications (PTMs). Across both SAS and MDA‐MB‐231 cells, immunoprecipitated PKM2 showed no significant changes in acetylation, pS37, pY105, or *O*‐GlcNAcylation between WT and F26W at 24 h, with or without erastin treatment (Figure 6e). No appreciable changes in the ubiquitination of PKM2 were observed between WT and F26W (Figure 6e). Similarly, no appreciable differences were detected at 12 h (Figure S9c, Supporting Information). These results suggest that under these conditions, compensatory regulation via these PTMs may be limited, and erastin‐induced GSH depletion does not appear to have a significant impact on the PTM landscape of PKM2.

We further evaluated functional consequences by measuring pyruvate kinase activity and lipid ROS in PKM2 WT‐ and F26W‐expressing cells treated with or without erastin at 24 h. Erastin reduced pyruvate kinase activity in WT‐expressing cells but not in F26W (Figure 6f), while lipid ROS accumulation was consistently higher in WT compared with F26W under the same conditions (Figure 6g). It is noted that the magnitude of induction was consistently smaller in F26W than in WT (Figure 6g).

Together, these results demonstrate that impaired GSH binding, rather than compensatory PTM regulation, is the principal determinant of PKM2 activity and ferroptotic sensitivity under erastin. F26 is therefore critical for GSH‐mediated allosteric activation of PKM2. Disruption of this residue decouples PKM2 from redox control and alters ferroptosis susceptibility without impairing canonical activation by FBP. Collectively, our findings support a model in which PKM2 acts as a metabolic switch that regulates cancer cell susceptibility to ferroptosis under GSH‐limited conditions, suggesting that targeting PKM2 activation in the context of compromised antioxidant defenses may represent a therapeutic strategy for cancers with high PKM2 and SLC7A11 expression.

### 2.7 Metabolic and Lipidomic Profiling Reveals Metabolic Reprogramming Induced by PKM2 Activation in GSH‐Depleted Cells

**Figure 7:**
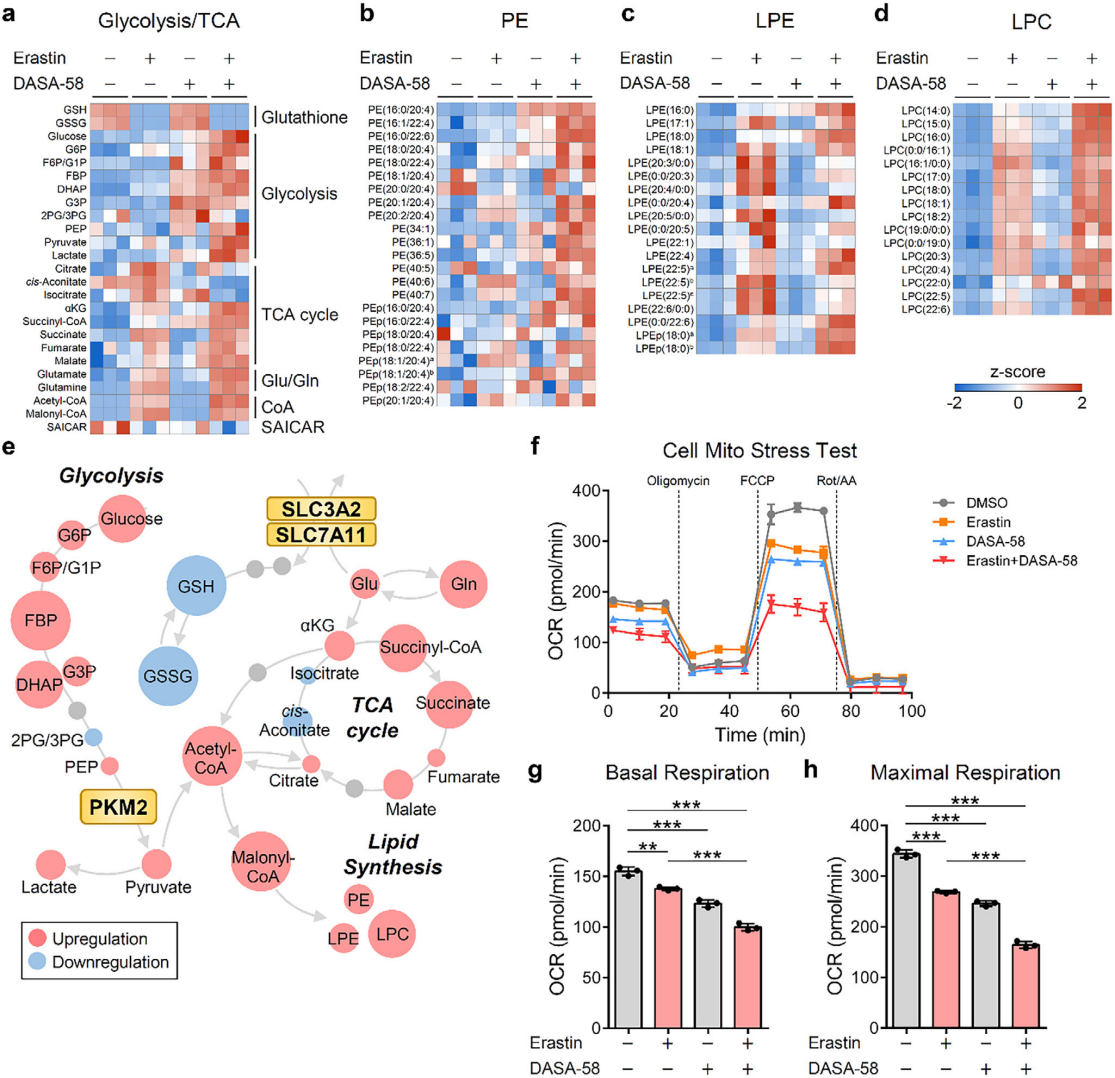
Metabolomic reprogramming and mitochondrial respiration changes induced by erastin and DASA‐58 in MDA‐MB‐231 cells. a‒d) Heatmaps showing the abundance of central carbon metabolites (a), PE species (b), LPE species (c), and LPC species (d). MDA‐MB‐231 cells were treated with the effector combinations (10 µм erastin/20 µм DASA‐58) for 24 h. Metabolite levels were normalized to total protein levels (*n* = 3). G1P, glucose 1‐phosphate; G3P, glyceraldehyde 3‐phosphate; 2PG/3PG, 2‐/3‐phosphoglyceric acid; αKG, α‐ketoglutarate. The superscripts indicate isomers that are indistinguishable. e) Schematic representation of the metabolic changes induced by erastin and DASA‐58 co‐treatment. Upregulated and downregulated metabolites, compared to the untreated control, are indicated in red and blue, respectively. Circle sizes correspond to the magnitude of change, with key pathways affected by the combination treatment highlighted. Glu, glutamate; Gln, glutamine. f) OCR measured in a Cell Mito Stress Test. MDA‐MB‐231 cells were treated with the indicated compounds (10/20 µм DASA‐58) for 12 h before the measurement (*n* = 3). FCCP, carbonyl cyanide 4‐(trifluoromethoxy)phenylhydrazone. Rot/AA, rotenone/antimycin A. g‒h) Analysis of mitochondrial respiration parameters from (f): basal respiration (g) and maximal respiration (h) (*n* = 3). Data are shown as mean ± SD for (f)‒(h). One‐way ANOVA with Tukey's multiple comparison test for (g) and (h). ^**^ *p* < 0.01, ^***^ *p* < 0.001.

To elucidate the metabolic alterations underlying the enhanced ferroptosis sensitivity upon PKM2 activation in GSH‐depleted cells, we performed metabolomic and lipidomic analyses on MDA‐MB‐231 and SAS cells treated with erastin, DASA‐58, or their combination. The heatmaps of central carbon metabolites (**Figure** **7**a; Figure S10a, Supporting Information) revealed significant changes in glycolytic and TCA cycle intermediates. Key glycolytic metabolites, glucose‐6‐phosphate (G6P), F6P, and pyruvate, were markedly elevated following erastin treatment, with further increases observed upon co‐treatment with DASA‐58. These elevations indicate an upregulation of glycolytic flux, likely due to PKM2 activation under GSH‐depleted conditions. Significant reductions in GSH and its oxidized form, GSSG, were observed with erastin and erastin‐DASA‐58 treatments, confirming the disruption of glutathione‐dependent redox homeostasis. Concurrent increases in glutamate and glutamine levels suggest impaired SLC7A11 function due to erastin's inhibition of cystine uptake. The dramatic upregulation of acetyl‐CoA under the combination treatment implies a metabolic shift favoring lipid synthesis and other anabolic processes. SAICAR abundance was essentially unchanged across all conditions, unlike glycolytic intermediates and several TCA cycle metabolites (Figure 7a; Figure S10a, Supporting Information).

Analysis of lipid metabolism (Figure 7b‒d; Figure S10b‒d, Supporting Information) showed substantial alterations in membrane lipid composition. We observed elevations in phosphatidylethanolamines (PE), PE plasmalogens (PEp), and lysophosphatidylethanolamines (LPE) species following erastin or erastin‐DASA‐58 treatment. These lipid species are known substrates for lipid peroxidation, and their accumulation may contribute to the ferroptotic process.^[^ ^46^, ^47^ ^]^ Heatmaps for lysophosphatidylcholines (LPC) also displayed significant shifts, indicating extensive remodeling of lipid metabolic pathways. These lipidomic changes suggest that the combination treatment disrupts membrane integrity and promotes ferroptosis through enhanced lipid peroxidation.^[^ ^26^ ^]^

Analysis of SAICAR abundance revealed that intracellular SAICAR levels remained essentially unchanged across all PKM2 variants and treatment conditions (Figure S10e, Supporting Information). In *PKM*‐depleted SAS cells reconstituted with WT, F26W, or Q393K, the F26W line showed a small but significant increase in basal SAICAR, whereas Q393K did not differ significantly from WT. In MDA‐MB‐231 cells, both F26W and Q393K‐repexpressing cells showed modestly elevated baseline SAICAR compared to WT. Notably, erastin treatment has a limited or no effect on SAICAR levels in any of the variants. These results align with the low cellular concentration and its moderate affinity for PKM2.^[^ ^36^, ^48^ ^]^

A schematic diagram (Figure 7e) summarizes the metabolic alterations induced by erastin and DASA‐58 co‐treatment, highlighting the interplay between glycolysis, lipid metabolism, and oxidative stress pathways. GSH depletion by erastin impairs antioxidant defenses, while PKM2 activation by DASA‐58 enhances glycolytic flux, leading to increased pyruvate production and ROS generation. The resultant oxidative stress, coupled with altered lipid metabolism, sensitizes cancer cells to ferroptosis.

To determine whether PKM2 activation affects its subcellular localization, we performed subcellular fractionation followed by Western blotting in SAS and MDA‐MB‐231 cells (Figure S10f, Supporting Information). No significant changes in PKM2 nuclear translocation were observed across treatments, indicating that the ferroptotic effects are primarily mediated through PKM2's cytoplasmic functions.

We evaluated mitochondrial function by measuring oxygen consumption rates (OCR) in treated cells (Figure 7f‒h; Figure S10g‒i, Supporting Information). Both erastin and DASA‐58 individually reduced basal respiration rates in MDA‐MB‐231 cells, with the combination treatment resulting in the lowest basal respiration (Figure 7g). Maximal respiration, indicative of mitochondrial capacity, was slightly decreased by each treatment alone but significantly attenuated by the combination (Figure 7h). Similar trends were observed in SAS cells, though statistical significance was limited (Figure S10h,i, Supporting Information).

We conducted a 13C_6_‐glucose labeling analysis in SAS and MDA‐MB‐231 cells. After treating the cells with erastin and/or DASA‐58 for 24 h, the cells were then labeled with 13C_6_‐glucose for 30 min. The metabolomic characterization revealed efficient labeling (> 90%) of cellular glucose (Figure S10j, Supporting Information). We then evaluated the level of acetyl‐CoA, the key molecule bridging glycolysis and the TCA cycle, and the building block of fatty acids and lipids. We observed that ≈50% of acetyl‐CoA was labeled in each group (Figure S10k, Supporting Information). The total level of acetyl‐CoA dramatically increased under erastin treatment. While no significant difference in the labeling efficiencies, the total amount of acetyl‐CoA was further upregulated in the erastin/DASA‐58 co‐treated groups, which is consistent with our targeted metabolomic results. This result indicated that the co‐treatment of erastin and DASA‐58 indeed enhanced the central carbon metabolism, facilitating lipid metabolism through the accumulation of acetyl‐CoA.

Transcriptomic analysis of MDA‐MB‐231 cells treated with erastin and DASA‐58 revealed significant enrichment of gene sets related to ferroptosis, the unfolded protein response, and amino acid metabolism (Figure S11a, Supporting Information). Notably, key ferroptosis‐related genes, including *SLC7A11*, *SLC3A2*, and *GCLM*, were markedly upregulated following erastin treatment, supporting the role of glutathione metabolism in ferroptotic regulation (Figure S11b, Supporting Information). In contrast, genes involved in glycolysis and lipid metabolism exhibited only moderate changes, suggesting that ferroptosis induction is the dominant cellular response under these conditions. These results suggest that PKM2 activation in GSH‐depleted cells exacerbates mitochondrial dysfunction, primarily due to increased ROS production and lipid peroxidation, leading to mitochondrial membrane damage and impairing the electron transport chain. This disruption in mitochondrial respiration significantly sensitizes cancer cells to ferroptosis, reinforcing the metabolic vulnerability of PKM2‐dependent tumors under oxidative stress conditions.

### 2.8 Combined Targeting of PKM2 and SLC7A11 Enhances Ferroptosis Sensitivity and Suppresses Tumor Growth

**Figure 8:**
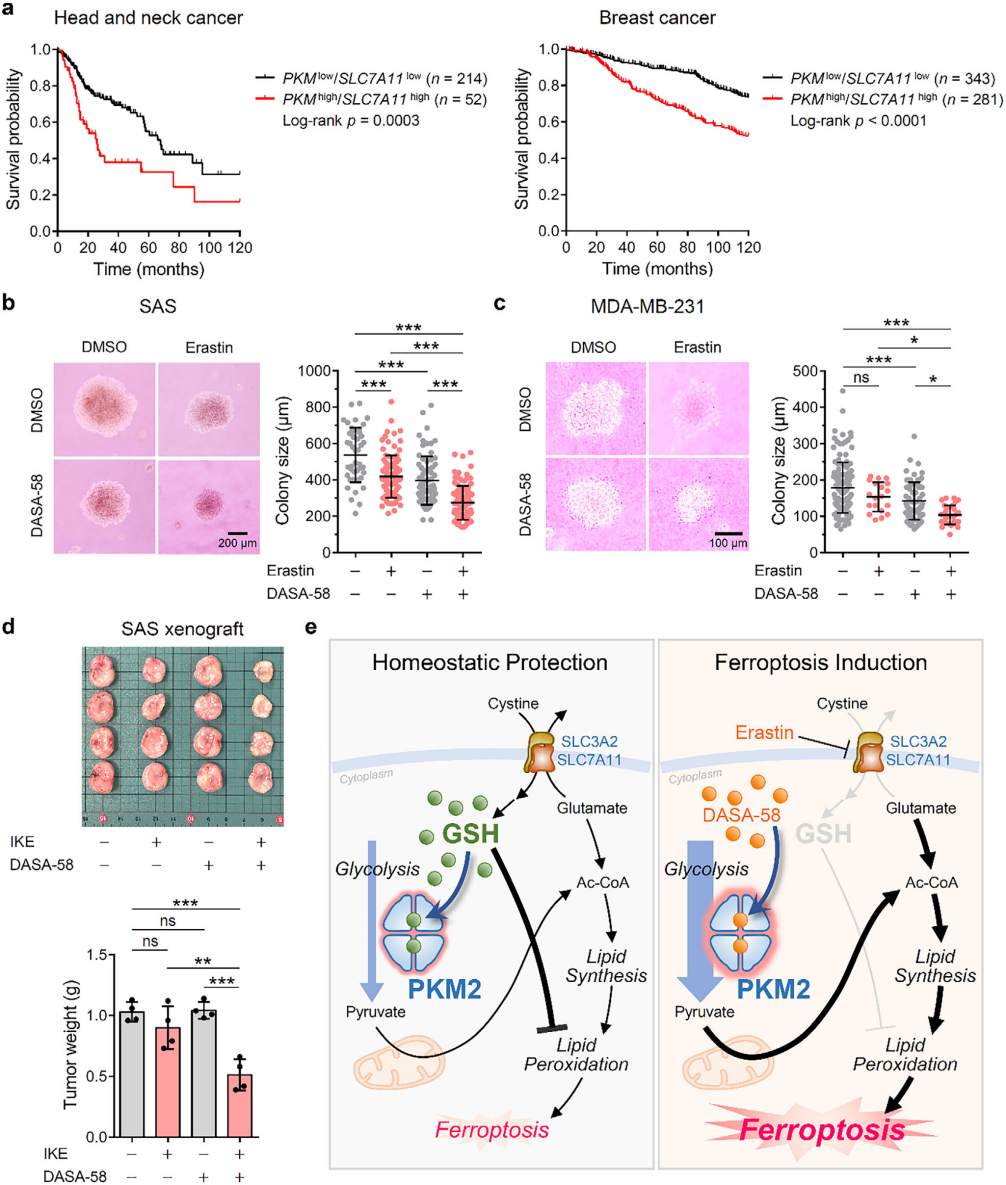
Survival analysis, colony formation, tumor growth, and a mechanistic model of PKM2 activation and GSH depletion in cancer therapy. a) Kaplan‐Meier survival analysis for patients in HNSCC (TCGA Pan‐Cancer) and breast cancer (METABRIC) cohorts based on *PKM* and *SLC7A11* levels. Optimal cutoff values for *PKM* and *SLC7A11* were determined using Youden's index. b, c) Soft agar colony formation assay with SAS (b) and MDA‐MB‐231 (c) cells. Cells were embedded in an agarose matrix and treated with various effector combinations (SAS: 2 µм erastin and/or 1 µм DASA‐58 for 1 week; MDA‐MB‐231: 2 µм erastin and/or 10 µм DASA‐58 for 2 weeks). Representative images of the colonies are shown on the left, and quantification of colony size (greater than 50 µm) is presented on the right. d) Mouse xenograft model. SAS cells were subcutaneously injected into nude mice. After one week, mice received peritumoral injections of combined effectors [50 mg kg^−1^ IKE and/or 50 mg kg^−1^ DASA‐58] three times per week. Images of the dissected tumors are shown (upper panel), with quantification of tumor weight presented in the lower panel (*n* = 4). e) Proposed working model for the study. This schematic summarizes the cooperative effects of erastin and DASA‐58 on PKM2 activation, GSH depletion, and ferroptosis induction. It highlights how these treatments drive metabolic reprogramming and increase lipid peroxidation, facilitating ferroptotic cell death. Ac‐CoA, acetyl‐CoA. Data are presented as mean ± SD for (b)‒(d). One‐way ANOVA with Tukey's multiple comparison test for (b)‒(d). ^*^ *p* < 0.05, ^**^ *p* < 0.01, ^***^ *p* < 0.001, ns: *p* > 0.05.

Analysis of public patient cohort databases revealed that high expression levels of both *PKM* and *SLC7A11* are significantly associated with poor survival outcomes in patients with HNSCC and breast cancer (Figure S12a,b, Supporting Information). Specifically, patients exhibiting elevated expression of both genes had markedly lower overall survival rates compared to those with low expression levels (**Figure** **8**a). This correlation suggests the clinical relevance of *PKM* and *SLC7A11* in cancer progression and supports the proposal that co‐targeting these molecules could be a viable therapeutic strategy.

To test this potential, we conducted soft agar colony formation assays using SAS and MDA‐MB‐231 cells. Treatment with either erastin or DASA‐58 individually resulted in modest reductions in colony size (Figure 8b,c). Notably, the combination treatment led to significant suppression of colony formation, indicating that PKM2 activation enhances the susceptibility of GSH‐depleted cancer cells to ferroptosis, inhibiting anchorage‐independent growth.

To assess the in vivo relevance of our findings, we employed a mouse xenograft model using SAS cells. We utilized imidazole ketone erastin (IKE), a metabolically stable analog of erastin with similar biological activity (Figure S12c, Supporting Information).^[^ ^49^ ^]^ Mice bearing SAS tumors were treated with vehicle control, IKE, DASA‐58, or a combination of IKE and DASA‐58 over ≈5 weeks following subcutaneous tumor implantation (Figure S12d, Supporting Information). Throughout the treatment period, no significant changes in body weight were observed across all groups (Figure S12e, Supporting Information), indicating good tolerability. Tumors in mice treated with both IKE and DASA‐58 showed significantly reduced growth compared to control or single‐agent treatments (Figure 8d; Figure S12f, Supporting Information). We also performed this series of experiments using MDA‐MB‐231 xenografts, which exhibited significant growth inhibition when treated with the combination therapy (Figure S12g‒i, Supporting Information).

This substantial suppression of tumor growth by the combination treatment suggests that simultaneous modulation of PKM2 activity and GSH levels profoundly impacts tumor progression. The metabolic flux alterations observed with this combination treatment suggest that PKM2 activation in the context of low GSH disrupts cancer cells' energy production and redox homeostasis, leading to enhanced oxidative stress and lipid peroxidation. This shift may represent a broader vulnerability in tumors with upregulated glycolysis and redox protection mechanisms (Figure 8e).

## 3 Discussion

Our study uncovers that GSH acts as an endogenous A‐A type allosteric activator of PKM2, stabilizing the enzyme in its highly active tetrameric form. This discovery identifies a previously unrecognized PKM2‐GSH axis that serves as a critical intersection between cancer metabolism and redox homeostasis. By enhancing PKM2 activity, GSH promotes glycolytic flux, ensuring a steady supply of ATP and metabolic intermediates essential for anabolic processes and cell survival. In cancer contexts where GSH levels are elevated to counteract oxidative stress,^[^ ^50^ ^]^ this interaction maintains PKM2 in an active state, supporting the metabolic and biosynthetic demands of rapidly proliferating tumors. Furthermore, PKM2 activity influences the availability of NADPH through the PPP, aiding in GSH regeneration.^[^ ^51^ ^]^ This feedback loop connects glycolysis with redox homeostasis, enabling cancer cells to dynamically switch between metabolic states and adapt to hostile environments.

PKM2 is regulated by both metabolic and non‐metabolic mechanisms, allowing it to integrate various cellular signals. While FBP is a known allosteric activator of PKM2,^[^ ^9^ ^]^ our study uniquely identifies GSH as another allosteric activator that enhances glycolytic flux. Although PKM1 is not allosterically responsive to GSH, both isoforms nonetheless modulate NADPH production and GSH regeneration by differentially directing G6P either toward pyruvate (via PKM1/2 activity) or the PPP, respectively.

Structurally, our analyses show that K311 and N350 from each subunit form key hydrogen bonds with GSH at the A‐A interface. Interestingly, F26 tightly packs against GSH via van der Waals contacts and acts as the critical allosteric “anchor.” Substitution of F26 with tryptophan (F26W) sterically impedes this interaction and reduces GSH‐mediated activation of PKM2 both in vitro and in cells, pinpointing F26 as the critical residue for GSH engagement. These findings further suggest that, under GSH‐depleted conditions, PKM2 may become subject to alternative regulatory mechanisms, such as PKM2 acetylation, ubiquitination, phosphorylation, and *O*‐GlcNAcylation to modulate its non‐canonical functions in cancer.^[^ ^52^ ^]^

Non‐metabolic regulation includes somatic mutations that alter its enzymatic activity and interactions with nuclear transporters like importin α5 and KDM8, facilitating its translocation into the nucleus, where it functions as a transcriptional co‐activator influencing gene expression.^[^ ^9^, ^13^, ^53^, ^54^ ^]^ These diverse regulatory mechanisms suggest that PKM2 is vital to coordinating metabolic processes with cellular proliferation and survival pathways, contributing to tumorigenesis and cancer progression.

The dual role of the PKM2‐GSH axis is particularly significant in the IF region of tumors. Our transcriptomic analyses of matched HNSCC samples using WGCNA and MMI network identified PKM2 and SLC7A11 as central nodes within metabolic modules significantly upregulated in the IF region. These modules encompass glycolysis/gluconeogenesis, fatty acid metabolism, glutathione metabolism, and ferroptosis pathways, essential for maintaining energy production, biosynthesis, and redox balance in cancer cells at the tumor's leading edge, a region also associated with inflammation.^[^ ^43^ ^]^ The complementary spatial transcriptomic analysis further confirmed the co‐expression of PKM2, SLC7A11, glycolysis, and ferroptosis pathways at the IF region. The elevated expression of PKM2 and SLC7A11 at the IF suggests that cancer cells strategically exploit the PKM2‐GSH axis to facilitate the metabolic flexibility required for tumor progression and metastasis, supporting its potential as a therapeutic target in aggressive cancers.

Recent studies have further highlighted the interplay between lipid metabolism, epithelial‐mesenchymal transition (EMT), inflammation, and ferroptosis in malignant cancers, representing metabolic vulnerabilities that can be therapeutically targeted.^[^ ^43^, ^55^, ^56^ ^]^ ZEB1, an essential EMT transcriptional factor, induces the expression of lipogenic enzymes, leading to enhanced lipid synthesis necessary for membrane remodeling and energy storage in invasive cancer cells.^[^ ^56^ ^]^ This upregulated lipogenesis not only supports rapid proliferation but also impacts ferroptosis susceptibility. The increased abundance of polyunsaturated fatty acids in cell membranes makes cancer cells more prone to lipid peroxidation, a hallmark of ferroptosis.^[^ ^26^ ^]^ Targeting lipid metabolic pathways associated with EMT has shown promise. For instance, inhibiting fatty acid synthase in KRAS‐mutant lung cancer leads to ferroptosis and tumor suppression.^[^ ^55^ ^]^ Similarly, inhibiting stearoyl‐CoA desaturase and fatty acid desaturase 2 in high ZEB1‐expressing cancer cells effectively induces ferroptosis.^[^ ^56^ ^]^ These findings suggest that targeting lipid metabolism can overcome ferroptosis resistance mechanisms and induce cell death in aggressive cancers.

In this context, SLC7A11, a crucial component of the cystine/glutamate antiporter xCT, emerges as a potential biomarker for ferroptosis susceptibility and therapeutic targeting. Elevated SLC7A11 expression is a common adaptive mechanism in many cancers, sustaining high GSH levels to mitigate oxidative stress and resist cell death, particularly ferroptosis.^[^ ^27^ ^]^ Our data suggest that cancers with high SLC7A11 expression and high PKM2 expression are more reliant on the PKM2‐GSH axis for survival, making SLC7A11 a potential biomarker for identifying tumors that may respond to therapies targeting this metabolic vulnerability. Accumulated evidence supports the role of SLC7A11 as a biomarker and therapeutic target. High SLC7A11 expression has been found to correlate with poor prognosis in various cancers.^[^ ^57^ ^]^ Targeting SLC7A11 can sensitize cancer cells to ferroptosis and enhance the efficacy of conventional therapies.^[^ ^58^ ^]^ Additionally, SLC7A11 has been implicated in regulating the tumor microenvironment and influencing antitumor immunity, suggesting that SLC7A11 inhibitors could enhance immunotherapy responses.^[^ ^59^ ^]^

Mechanistically, our study demonstrates that the combined inhibition of SLC7A11 (leading to GSH depletion) and activation of PKM2 induces substantial alterations in central carbon metabolism and lipid remodeling, particularly affecting glycolysis, the TCA cycle, and lipid species composition. These metabolic changes disrupt the balance of energy production and redox homeostasis, culminating in ferroptotic cell death. The extensive reorganization of lipid pathways contributes to the accumulation of lipid peroxides, further promoting ferroptosis. Notably, impaired mitochondrial function observed with this dual‐targeting approach indicates a significant impact on cellular bioenergetics, adding another layer to the metabolic vulnerabilities that can be therapeutically exploited.

To ensure that our findings are directly relevant to the biology of HNSCC, we selected the SAS cell line, which is derived from the tongue and represents the dominant oral cavity subtype in HNSCC, accounting for ≈90% of cases.^[^ ^60^ ^]^ We then validated the role of the PKM2‐GSH‐ferroptosis pathway in MDA‐MB‐231 breast cancer cells and further explored our observations in U87 glioblastoma and H1299 lung carcinoma cell lines. This indicates that GSH‐dependent activation of PKM2 can sensitize various tumor types to ferroptosis. Additionally, pan‐cancer analyses from TCGA and DepMap displayed a consistent correlation between the expression levels of *PKM* and *SLC7A11* and sensitivity to the drug erastin. We utilized both clinical and spatial transcriptomic assessments, along with data from the TCGA‐HNSC dataset, which serves as the most comprehensive public resource for HNSCC. This highlights the clinical relevance of our biochemical insights to various types of solid tumors.

In conclusion, our study elucidates the PKM2‐GSH‐SLC7A11 axis that modulates glycolysis, redox homeostasis, lipid metabolism, and ferroptosis, essential in tumors with high PKM2 and SLC7A11 expression. By simultaneously depleting GSH and activating PKM2, we disrupt cancer cells' metabolic adaptations, driving them toward ferroptotic cell death. The identification of SLC7A11 as a potential biomarker further enhances the translational potential of our findings, as it could guide patient stratification and therapeutic decision‐making. This dual‐targeting strategy is particularly compelling for tumors at the invasive front, where metabolic reprogramming is crucial for survival and proliferation. Our findings propose a promising intervention for targeting aggressive tumors by exploiting their metabolic vulnerabilities associated with the PKM2‐GSH axis and lipid metabolism linked to EMT, advancing therapeutic strategies against malignant cancers.

## 4 Experimental Section

### Cancer Cell Model

SAS (RRID: CVCL_1675; a gift from Dr. Muh‐Hwa Yang^[^ ^43^ ^]^ at National Yang Ming Chiao Tung University, Taiwan), MDA‐MB‐231 (RRID: CVCL_0062; ATCC, HTB‐26), and U87 (RRID: CVCL_0022; Bioresource Collection and Research Center, Taiwan, 60360) cells were cultured in Dulbecco's Modified Eagle Medium (DMEM, Gibco, 12100046) supplemented with 10% fetal bovine serum (FBS, Cytiva). H1299 (RRID: CVCL_0060; ATCC, CRL‐5803) cells were cultured in Roswell Park Memorial Institute (RPMI) 1640 Medium (Gibco, 31800022) supplemented with 10% FBS. Cells were maintained in a humidified incubator with a 5% CO_2_ atmosphere at 37 °C and routinely screened for mycoplasma contamination using PCR‐based detection.

### Xenograft Mouse Model

The animal study protocol was reviewed and approved by the Institutional Animal Care and Use Committee of National Tsing Hua University (approval number: NTHU‐IACUC‐112027). The animal study was carried out under the institutional guidelines and met the animal welfare standards. SAS cells (1 × 10^6^ cells) and MDA‐MB‐231 cells (5 × 10^6^ cells) were suspended in 50 µL of sterile PBS and mixed with an equal volume of Matrigel Matrix (BD, Franklin Lakes, NJ, USA). The mixture was kept on ice and subcutaneously implanted into the back flanks of 4‐week‐old female nude mice. Mice were maintained in the specific pathogen‐free area of the animal facility (NTHU). Drug administration started ≈1 or 2 weeks for SAS and MDA‐MB‐231 cells, respectively, after the implantation, when the average tumor size reached 100 mm^3^. Vehicle (PBS), IKE (50 mg kg^−1^), DASA‐58 (50 mg kg^−1^), or their combinations were suspended in 100 µL sterile PBS and delivered to the mice three or two times a week for SAS and MDA‐MB‐231 cells, respectively, via peritumoral injection. Tumor sizes [size (mm^3^) = length × width^2^ × 0.5] and mouse body weights (g) were measured. After 5 to 6 weeks of treatment, the mice were sacrificed, and the tumors were dissected, weighed, and pictured.

### Plasmid Construction

Primers were synthesized by Mission Biotech, Taiwan. For recombinant protein purification, full‐length PKM2 and PKM1 cDNAs were PCR‐amplified (HiFi DNA polymerase) and inserted into the pET28a vector (Novagen) through restriction enzyme digestion and ligation procedures. The PKM2 variants (C358A, F26W, Q393K, K311R, and L353W) were generated by the site‐directed mutagenesis method. For the EGFP‐PKM2 fusion construct, an extra EGFP cDNA was added to the N‐terminus of the PKM2 coding region and in‐frame with it. For the cell‐based expression of 6xHis‐PKM2, the His‐tagged PKM2 cDNA was PCR‐amplified and inserted into the pcDNA‐HA vector. Constructed plasmids were stained with Novel Juice (GeneDireX, Las Vegas City, NV, USA), checked by agarose gel electrophoresis, and verified by Sanger sequencing.

### Protein Purification

*Escherichia coli* BL21 (DE3) competent cells were transformed with the plasmids carrying the PKM2, EGFP‐PKM2, or PKM1 target sequence and cultured in Luria broth. Protein expression was achieved by adding isopropyl‐β‐d‐thiogalactopyranoside to 1 mм under 16 °C incubation for 21 h. The cells were harvested by centrifugation and resuspended in Buffer A (50 mм Na_2_HPO_4_, pH 7.5, 100 mм NaCl). After sonication and centrifugation, the crude protein extract was loaded into a column containing cobalt‐chelated TALON Metal Affinity Resin (Clontech) pre‐equilibrated with Buffer A. After affinity interaction with the 6xHis tag, the column was washed with Buffer A containing 10–40 mм imidazole to remove non‐specific interactions. The target protein was then eluted with Buffer A containing 150 mм imidazole. Imidazole was then removed by dialysis using an Amicon Ultra Centrifugal Filter unit (30 kDa cutoff, Millipore). Protein purity was checked by SDS‐PAGE, followed by Coomassie Brilliant Blue staining. Protein concentration was determined by the Bradford assay (Protein assay dye, Bio‐Rad).

### Pyruvate Kinase Activity Assay and Kinetic Analysis

The enzymatic activity of pyruvate kinase was determined by an LDH‐coupled reaction. For purified recombinant PKM2, a steady‐state kinetic reaction was carried out in a buffer background of 50 mм Tris (pH 7.5) and 100 mм KCl. Recombinant PKM2 (25 ng) was incubated with 5 mм MgCl_2_, 1 mм ADP, 0.4 mм NADH, 2 U LDH, and 1 mм PEP in a 200‐µL reaction mixture at 37 °C, and OD_340_ was measured over a 10‐min period. The activity of PKM2 was calculated from the initial rate of the reaction. For the cell‐based pyruvate kinase activity assay, total cell lysate was prepared for the activity assay. To determine the coupling effect of an allosteric effector, the dissociation constants of PEP, *K_a_* [PEP], defined as the PEP concentration which gives rise to 1/2*V_max_* reaction rate was determined at different concentrations of GSH (0 to 1 mм) and FBP (0 to 2 mм) using the Michaelis‐Menten equation. The coupling constant (*Q_ax_*) indicating the effect of an allosteric effector on PEP binding is calculated from the linked‐function analysis $\;K_{a}=K_{ia}^{0}\left[(K_{ix}^{0}+\left[\mathrm{PEP}\right]\right)/K_{ix}^{0}+Q_{ax}\left[\mathrm{PEP}\right])]$, where $K_{ia}^{0}$ represents the dissociation constant for PEP in the absence of an allosteric effector, and $K_{ix}^{0}$ represents the dissociation constant for an allosteric effector in the absence of PEP.^[^ ^38^ ^]^ The kinetic measurements were done using the CLARIOstar Plus microplate reader (BMG LABTECH).

### MST Assay

EGFP‐tagged PKM2 was purified, dialyzed, and diluted to 100 nм with the assay buffer (50 mм Na_2_HPO_4_, pH 7.5, 100 mм NaCl, 0.05% Tween‐20). Freshly prepared GSH was serially diluted with the assay buffer and mixed with the 100‐nм EGFP‐PKM2 solution in a 1:1 volume ratio. The final GSH concentration ranged from 85 nм to 550 µм, including a 0‐µм control. The samples were incubated at room temperature for 1 h (light avoided) and loaded into Monolith Premium Capillaries (NanoTemper Technologies). MST assay was carried out using the Monolith NT.115 Blue/Red instrument (NanoTemper Technologies) at 20% excitation power and High MST power. The dissociation constant was derived by fitting the experimental data to the one‐site‐specific binding model (GraphPad PRISM 7).

### Blue Native PAGE

Purified recombinant PKM2 (3 µg) was incubated with the indicated ligand concentration in a 30‐µL reaction mixture. The background buffer comprised 50 mм Na_2_HPO_4_ (pH 7.5), 100 mм NaCl, and 10% glycerol. After 1 h of incubation at 37 °C, the samples were subjected to blue native PAGE by using the XCell SureLock Mini‐Cell electrophoresis system (Invitrogen) and the NativePAGE 3–12% Bis‐Tris Mini Protein Gels (Invitrogen). NativeMark Unstained Protein Standard (Invitrogen) was used as the protein marker. The cathode buffer comprised 50 mм tricine, 7.5 mм imidazole (pH 7.0), and 0.02% Coomassie blue G‐250. The anode buffer was composed of 25 mм imidazole (pH 7.0). Electrophoresis was carried out at 4 °C. After halfway through the electrophoresis, the cathode buffer was exchanged for the same buffer with only 0.002% Coomassie blue G‐250 to remove excess dye. When the dye front reached the bottom of the gel, the electrophoresis was terminated, and the PKM2 protein was visualized by Coomassie Brilliant Blue R‐250 staining.

### Protein Crystallization

Purified recombinant PKM2 was diluted to 8 mg mL^−1^ and incubated with 5 mм GSH and 2 mм FBP on ice in the background buffer of 40 mм Tris (pH 8.0) and 100 mм NaCl. The protein sample was then mixed with the reservoir solution (0.2 м sodium tartrate, 0.1 м KCl, 20% PEG3350) in a 1:1 volume ratio in a hanging drop vapor diffusion setup using the 24‐well VDX plates (Hampton Research). Protein crystals were observed after ≈1 week of incubation at 4 °C.

### X‐ray Diffraction Data Collection and Model Building

X‐ray diffraction data were collected at the BL13B1 beamline at the National Synchrotron Radiation Research Center (NSRRC, Hsinchu, Taiwan). PKM2 crystals were freshly mounted onto a goniometer, and data were collected using an ADSC Quantum 315r CCD area detector. Dataset integration and scaling were done by using HKL‐2000. The output SCA file was transformed into an MTZ file using the scalepack2mtz program (CCP4i 7.1.015). The phasing process was done using a previous PKM2 structure (PDB: 4B2D) as the template model and the molecular replacement method (Molrep, CCP4i). Structure refinement was done using the refmac5 program (CCP4i) and the Coot 0.9.4 program. The RMSD values between structures were calculated by the “Superpose PDB files” module of Phenix 1.19.2‐4158. The highly flexible B domains were removed prior to RMSD calculation. The phylogenetic tree of structures was generated by MEGA 11.0.13. Structural illustration was done using PyMOL.

### Molecular Docking Analysis

The PKM2‐SAICAR docking model was prepared by using Discovery Studio Client v22.1.0.21297 (BIOVIA). In brief, the PKM2 model (PDB: 3SRD) and the SAICAR model were prepared, and the activator binding site was selected as the binding pocket. The SAICAR molecule was then docked against the selected pocket using the CDOCKER algorithm. The docked pose with the lowest CDOCKER energy was selected as the docking result.

### GSH Competition Assay

Cobalt‐chelated TALON Metal Affinity Resin (Clontech) was equilibrated in the binding buffer: 50 mм Na_2_HPO_4_, pH 7.5, 100 mм NaCl, 0.1% NP‐40. Purified recombinant PKM2 protein (200 µg) was incubated with the resin (10 µL) and 2.5 mм GSH in the binding buffer for 30 min on ice. After removing the unbound substances and washing, the binding buffer alone or 5 mм SAICAR or FBP was incubated with the resin for 1 h at room temperature. After another washing step, the GSH remaining complexed with PKM2 on the resin was measured using the GSH‐Glo Glutathione Assay kit (Promega, V6911) following the manufacturer's instructions. A sample group without PKM2 was used as the background control.

### Cell‐Based PKM2 Pull‐Down Assay

HEK293T cells were transfected with pcDNA‐HA‐PKM2‐His plasmid using PolyJet In Vitro DNA Transfection Reagent (SignaGen Laboratories, SL100688), followed by DMSO or erastin treatment for 24 h. Cells were harvested, suspended in the lysis buffer (PBS with 0.1% NP‐40), and lysed by sonication. After removing cell debris by centrifugation, the lysates were subjected to 6xHis‐tag affinity binding by using cobalt‐chelated TALON Metal Affinity Resin (Clontech, 635503) at 4 °C for 30 min. The supernatant was removed, and the resin was washed with the lysis buffer. The PKM2‐complexed GSH on the resin was measured using the GSH‐Glo Glutathione Assay kit (Promega, V6911) following the manufacturer's instructions.

### GSH Measurement

Intracellular GSH levels were determined by using the GSH‐Glo Glutathione Assay kit (Promega, V6911) following the manufacturer's instructions. Briefly, cells were seeded at a density of 5000 to 10 000 cells per well in 96‐well tissue culture plates. After the indicated treatment, cell samples were processed according to the assay protocol and loaded into a 96‐well flat‐bottom white plate. The luminescence signal was integrated from a 0.5‐s interval by using the CLARIOstar Plus microplate reader (BMG LABTECH). GSH levels were normalized to cell numbers.

### Western Blotting

Cells on tissue culture plates were washed with ice‐cold PBS and then harvested by using cell scrapers. The cell pellet was then resuspended in the lysis buffer (50 mм Tris, pH 7.4, 150 mм NaCl, 0.5% NP‐40) supplemented with protease inhibitor cocktail (Thermo Fisher Scientific, 78430) and then homogenized using the Qsonica S‐4000 Ultrasonic Sonicator (Misonix) equipped with a cup horn. After centrifugation (13 000 rpm, 4 °C, 10 min), an appropriate amount of clear lysate was mixed with SDS loading dye and heated to 100 °C for 10 min. The samples were subjected to SDS‐PAGE with 10% acrylamide gels. The protein was then transferred onto an Amersham Protran 0.45 µm Nitrocellulose Blotting Membrane (Cytiva, 10600002) and blocked with 3% non‐fat dry milk in Tris‐buffered saline containing 0.1% Tween‐20 (TBST) for 1 h. After washing with TBST, the membrane was incubated overnight at 4 °C with primary antibodies at the appropriate dilutions. After washing with TBST, the membrane was then incubated with secondary antibodies at appropriate dilutions at room temperature for 3 h. After washing with TBST, the images were acquired by iBright 1500 imaging system (Invitrogen). The primary antibodies used in this study include: anti‐PKM2 [Cell Signaling Technology (CST), 4053S)], anti‐SLC7A11 (CST, 12691S), anti‐β‐actin (NOVUS, NB600‐501), anti‐6xHis (Cusabio, CSB‐MA000011M0m), anti‐HA (CST, 3724S), anti‐lamin B1 (Elabscience, E‐AB‐40257), anti‐α‐tubulin (GeneTex, GTX628802), anti‐acetylated lysine (CST, 9441S), anti‐PKM2 pS37 (Affinity Biosciences, AF7231), anti‐PKM2 pY105 (CST, 3827S), anti‐*O*‐GlcNAc (BioLegend, 838004), and anti‐ubiquitin (CST, 3936S).

### DepMap Analysis

The data analysis was based on the DepMap Public 24Q2 dataset downloaded from the DepMap portal (https://depmap.org/portal/). Gene expression level was downloaded in Log_2_(TPM + 1) format (Expression Public 24Q2), and GSH abundance was downloaded in Log_10_ scale format (Metabolomics). *PKM* gene effect data were downloaded from the CRISPR (DepMap Public 24Q2+Score, Chronos) dataset. Erastin sensitivity data were downloaded as AUC values [Drug sensitivity AUC (CTD^2)]. Spearman's correlation coefficients and the corresponding *p*‐values were calculated using Microsoft Excel formulas. Linear regression was done by using GraphPad PRISM 7.

### TCGA Pan‐Cancer Analysis

The TCGA Pan‐Cancer Atlas datasets were downloaded through cBioPortal (https://www.cbioportal.org/). The human gene sets (MSigDB v2024.1.Hs) used for enrichment analysis were downloaded from the Molecular Signature Database (https://www.gsea‐msigdb.org/gsea/msigdb/index.jsp). The ssGSEA analysis was done by using the ssGSEA module (v10.1.0) provided by the GenePattern platform (https://www.genepattern.org/). Spearman's correlation coefficients between gene sets and their corresponding *p*‐values were calculated using Microsoft Excel formulas. For the scattered dot plots of BRCA and HNSC, the ssGSEA scores were first Log_2_‐transformed, and the linear regression was done using GraphPad PRISM 7.

### WGCNA Analysis

To investigate the heterogeneous characteristics among the IC, IF, and M tumors of HNSCC, RNA‐seq data (GEO ID: GSE178537) from 65 samples in 21 HNSCC patients were retrieved and characterized.^[^ ^43^ ^]^ The RNA‐seq values for 18428 protein‐coding genes were normalized as Log_2_(TPM + 1), excluding genes with 90% of zero values among the 65 samples. WGCNA^[^ ^61^ ^]^ was performed using the R WGCNA package (v. 1.70‐3 in R 4.0.3). A soft‐thresholding power of 17 was selected to maximize the scale‐free topology model fit, which plateaued above 0.7 during the construction of the gene correlation network. Modules were identified using the “cutreeDynamic” function with deepSplit = 2 and minModuleSize = 5. Module characterization was performed using the “enrichGO” and “enrichKEGG” functions in the R ClusterProfiler package (v. 3.18.1 in R 4.0.3)^[^ ^62^ ^]^ based on GO biological process (BP)^[^ ^63^ ^]^ and KEGG pathway terms.^[^ ^64^ ^]^ To simplify the network for concise visualization, only genes associated with representatively enriched terms (one of the top 25; FDR‐*q* value ≤ 0.05) from the KEGG pathway (or GO BP) category and edges with correlation weights above 0.01 (i.e., co‐expressed gene pairs) were retained, preserving at most the top 50 edges for each node.

### MMI Network Analysis

To further examine differential expressions of subnetworks centered on modules, genes *PKM* and *SLC7A11* included, among the three tumor parts, previous works were followed^[^ ^65^, ^66^, ^67^ ^]^ to determine MMIs among the 172 identified modules. For each module *A*, the statistical significance of MMI for another module *B* based on the co‐expressed gene pairs between all genes of these two modules using the hypergeometric distribution was measured as follows:

(1) $$p=1-\sum_{i=0}^{m-1}\frac{N-M}{n-i}$$

where *m* and *n* are, respectively, the numbers of co‐expressed gene pairs and all possible gene pairs between all the genes of modules *A* and *B*. *M* and *N* are, respectively, the total numbers of all the co‐expressed gene pairs and all possible gene pairs between all the genes of module *A* and all the genes of the other modules, excluding module *A*. Note that the *p*‐values of module pairs *A*− *B* and *B*− *A* were separately computed, and an MMI between two modules was considered statistically significant when its *p*‐value for pair *A*− *B* (or *B*− *A*) was ≤ 0.05. This analysis identified 2279 MMIs among 168 modules, forming the MMI network.

Next, a subnetwork centered on the two modules containing genes *PKM* and *SLC7A11* and their neighboring modules was extracted. This subnetwork was trimmed by preserving only the top two modules most significantly enriched for each of the ten pathways, including citrate cycle (TCA cycle), cysteine and methionine metabolism, fatty acid elongation, fatty acid metabolism, ferroptosis, glutathione metabolism, glycolysis/gluconeogenesis, oxidative phosphorylation, pentose phosphate pathway, and pyruvate metabolism.

The gene correlation subnetwork centered on genes *PKM* and *SLC7A11* was then determined. Log_2_(tumor vs normal fold change) values of all genes in each module between tumors (T) from IC, IF, or M sites and corresponding normal tissues (N) were measured to quantify differential expression across the three tumor regions. Statistical analysis of differential expressions among the three tumor parts was conducted using a Wilcoxon signed‐rank test. Visualization of the gene correlation and MMI networks (or subnetworks) was performed using Cytoscape (version 3.10.2).^[^ ^68^ ^]^

### Spatial Transcriptomic Analysis

The spatial transcriptomic data set (GEO ID: GSE181300) was analyzed using Loupe Browser 8 (10x Genomics). The invasive front and inner tumor core regions were defined as previously described.^[^ ^43^ ^]^ The spatial expression pattern of genes (*PKM, SLC7A11, GCLM, and GCLC*) and gene sets (WP_FERROPTOSIS and HALLMARK_GLYCOLYSIS, MSigDB) were shown in Log_2_ scale.

### Ethics Approval and IHC Analysis

Informed written consent was obtained from all participants before enrollment. The tumor sample analysis was reviewed and approved by the Research Ethics Center for Human Subject Protection (REC) of National Yang Ming Chiao Tung University (approval number: 2022‐01‐019CC). The IHC analysis was carried out by using the Novolink Polymer Detection Systems (Leica Biosystems, RE7150‐K) following the manufacturer's instructions. Paraffin‐embedded HNSCC tumor sections were deparaffinized, rehydrated, and antigen retrieved with the citrate sodium buffer (pH 6.0). After blocking, the slides were stained with anti‐PKM2 (CST, 4053S) or anti‐GSH (abcam, ab19534) primary antibodies overnight. The slides were then incubated with horseradish peroxidase (HRP)‐conjugated polymer for 30 min, followed by diaminobenzidine development and Mayer's hematoxylin counterstaining. Images were acquired by using the Vectra Polaris Automated Quantitative Pathology Imaging System (AKOYA Biosciences).

### GEPIA2 Analysis

The Gene Expression Profiling Interactive Analysis 2 (GEPIA2, http://gepia2.cancer‐pku.cn/#index) server was used for correlation analysis using Spearman's correlation coefficient and the tumor versus normal comparison using TCGA and GTEx data.

### Cell Viability Assay

Cell viabilities were determined by the MTT (3‐[4,5‐dimethylthiazol‐2‐yl]‐2,5 diphenyl tetrazolium bromide) assay. Briefly, cells were seeded at a density of 5000 to 10 000 cells per well in 96‐well tissue culture plates. After the indicated treatment, the cell medium in each well was replaced with a 100‐µL medium containing 0.5 mg mL^−1^ MTT. The cells were incubated in a CO_2_ incubator for 2 h. The medium was removed, and 100 µL DMSO was added to each well. The absorbance at 570 nm was measured using the CLARIOstar Plus microplate reader (BMG LABTECH). For the cell death rescue experiments, cells were first pre‐treated with DMSO, 5 µм ferrostatin‐1, 20 µм Z‐VAD(OMe)‐FMK, or 20 µм necrostatin‐1 for 1 h in a complete culture medium. Then, the medium was exchanged into a culture medium containing DMSO, erastin, DASA‐58, or their combinations for 24 h, followed by the MTT assay.

### ROS Quantification

Cellular ROS levels were determined by using the H2DCFDA (Sigma, D6883) probe. Cells were first seeded into 6‐cm tissue culture plates (4 × 10^5^ SAS cells or 1 × 10^6^ MDA‐MB‐231 cells per plate). Effectors were added the next day as indicated. After 24 h of treatment, the cells were harvested and resuspended in a cell culture medium containing 10 µм H2DCFDA, incubated at 37 °C, and kept away from light for 30 min. The cells were then pelleted, PBS‐washed, and resuspended in PBS containing 0.1% NP‐40. The cells were then lysed using the Qsonica S‐4000 Ultrasonic Sonicator (Misonix) equipped with a cup horn. The lysates were loaded into a 96‐well flat‐bottom black plate. The ROS levels were indicated by the fluorescent intensities detected at 485/535 nm (excitation/emission) wavelengths by using the CLARIOstar Plus microplate reader (BMG LABTECH). The ROS levels were normalized to the total protein concentration of lysates determined by using the BCA Protein Assay Kit (Visual Protein, BC03‐500).

### Lipid Peroxidation Quantification

SAS (1 × 10^5^ cells/well) or MDA‐MB‐231 (2 × 10^5^ cells/well) were seeded onto glass slides inside a 6‐well plate. After effector treatment, the cells were stained with 1 µм C11 BODIPY in a cell culture medium for 15 min inside a CO_2_ incubator. The cells were then fixed with 3.7% paraformaldehyde at room temperature for 30 min in the dark. After PBS washing, the cells were covered with 100% glycerol and sealed onto a microscope slide with nail polish. After drying, the fluorescent images were acquired using the Apotome 3 microscope (Zeiss) with the GFP and DsRed channels. The fluorescent signals' intensities were quantified using the ZEN 2.3 blue edition software (Zeiss).

### Genetic Knockdown and Overexpression of Cancer Cells

*PKM* knockdown in cell models was achieved by using lentivirus transduction. To produce lentivirus carrying the desired short hairpin RNAs (shRNAs), a three‐plasmid system plus a control plasmid (pLKO) or a shRNA plasmid targeting the *PKM* gene was co‐transfected into HEK293T cells by using PolyJet In Vitro DNA Transfection Reagent (SignaGen Laboratories, SL100688) following the manufacturer's instructions. The cell culture medium containing the lentivirus particles was collected and filtered at 72 h post‐transfection. To knock down *PKM* gene expression, MDA‐MB‐231 or SAS cells were first seeded onto 6‐cm tissue culture plates. After cell attachment, lentivirus particles carrying pLKO control or a shRNA targeting the *PKM* gene were added to the cell culture medium supplemented with 8 µg mL^−1^ polybrene. Subsequent analyses were carried out at 48 h post‐transduction. To reconstitute PKM2 expression in *PKM*‐knockdown cells, the cells were transfected with the pcDNA‐HA‐PKM2 plasmid by using PolyJet In Vitro DNA Transfection Reagent (SignaGen Laboratories, SL100688).

### Immunoprecipitation and PTM Profiling

Cells expressing PKM2 WT and F26W were harvested from 10‐cm tissue culture plates and lysed in the lysis buffer (50 mм Tris, pH 7.4, 150 mм NaCl, 0.5% NP‐40) supplemented with protease inhibitor cocktail (Thermo Fisher Scientific, 78430) and Halt Phosphatase Inhibitor Cocktail (Thermo Fisher Scientific, 78428). The lysate was then homogenized using the Qsonica S‐4000 Ultrasonic Sonicator (Misonix) equipped with a cup horn. After centrifugation (13000 rpm, 4 °C, 10 min), the supernatant was allowed to incubate with anti‐HA (CST, 3724S) antibody and PureProteome Protein A/G Mix Magnetic Beads (Millipore, LSKMAGAG10) overnight at 4 °C. The beads bound to HA‐tagged PKM2 were collected using a magnetic rack, washed four times with lysis buffer, and eluted in SDS‐PAGE loading dye at 70 °C for 10 min. The eluates were analyzed by Western blotting with the indicated primary antibodies, and signals were detected using VeriBlot for IP Detection Reagent (HRP) (Abcam, 131366) and Trident Femto Western HRP Substrate (GeneTex, GTX14698).

### Targeted Metabolomic Analysis

Metabolomic analysis was performed as previously described.^[^ ^69^, ^70^ ^]^ Briefly, cells were plated in 6‐cm plates and subjected to the indicated treatment for 24 h. After rinsing with ice‐cold PBS, 1 mL of 80% methanol was added to each plate to precipitate protein. Cells were harvested with a cell scraper together with methanol. The samples (700 µL) were centrifuged at 12 000 × g, 4 °C for 10 min. The supernatant was dried with nitrogen gas. The residue was dissolved in 100 µL 50% acetonitrile. Samples were analyzed using Waters ultra‐high‐performance liquid chromatography coupled with Waters Xevo TQ‐XS Mass Spectrometry System (Waters Corp., Milford, MA, USA). The analysis was performed in negative and positive ion modes with multiple reaction monitoring (MRM). Major MRM fragment patterns of each analyte were determined using the tuning method. The optimized parameters were as follows: capillary voltage at 1 kV, desolvation temperature at 500 °C, source temperature at 150 °C, and gas flow at 1000 L h^−1^. The chromatographic separation was achieved on the Atlantis Premier BEH Z‐HILIC (100×2.1 mm, particle size of 1.7 µm; Waters Corp.) column at 30 °C with eluent A (water with 15 mм ammonium bicarbonate) and eluent B (90% acetonitrile with 15 mм ammonium bicarbonate), and the flow rate was set at 0.5 mL min^−1^.

### Lipidomic Analysis

Following the indicated treatment in 6‐cm plates, the cells were harvested with 1 mL of 80% methanol. The samples (300 µL) were mixed with 30 µL methanol and 900 µL methyl tert‐butyl ether (MTBE) and vortexed for 1 min, and then kept at room temperature for 1 h for protein precipitation. Samples were added to 156 µL of water, vortexed for 1 min, and kept at room temperature for 10 min. Samples were then centrifuged at 12 000 × g, 4 °C for 30 min, and 700 µL supernatant was transferred to a new microtube. The residual sample was extracted again with 700 µL MTBE and then kept at room temperature for 1 h, followed by centrifugation at 12 000 × g, 4 °C for 30 min, and 700 µl supernatant was pooled with the previous extract and dried with nitrogen gas. Before lipidomic analysis, samples were re‐dissolved in 1 mL isopropanol/acetonitrile/water (2:1:1, v/v/v) mixture followed by centrifugation at 12000 × g, 4 °C for 30 min. The supernatant was subjected to lipidomic analysis with ultra‐high‐performance liquid chromatography coupled with Xevo G2 XS (Waters Corp., Milford, MA, USA). The mass spectrometric analysis was operated in both positive and negative ion modes. The optimized parameters in both modes were as follows: capillary voltage at 1.5 kV, desolvation temperature at 550 °C, source temperature at 120 °C, and gas flow at 900 L h^−1^. The chromatographic separation was achieved on a BEH C18 column (100×2.1 mm, particle size 1.7 µm; Waters Corp., Milford, MA, USA) at 60 °C with elute A [acetonitrile/water (40:60, v/v) with 10 mм ammonium formate] and eluent B [isopropanol/acetonitrile (90:10, v/v) with 10 mм ammonium formate], and the flow rate was set at 0.45 mL min^−1^. The gradient profile was set as a linear gradient of 40%–99% B, 10 min. The column was then re‐equilibrated for 2 min for the next analysis. Samples for quality control (a mixture of all samples) were analyzed during the analytical runs after every 10 sample runs. All data were analyzed using Progenesis QI software (Nonlinear Dynamics, Newcastle, UK). The intensity of each mass ion was normalized with respect to the total ion count to generate a data matrix that included the retention time, *m*/*z* value, and the normalized peak area.

### 13C_6_‐Glucose Labeling Assay

Cells were plated in 6‐cm plates and subjected to the indicated treatment for 24 h. The culture medium was then replaced with fresh medium containing 25 mм 13C_6_‐glucose for 30 min. After rinsing with ice‐cold PBS, 1 mL of 80% methanol was added for protein precipitation. The samples were centrifuged at 12000 × g, 4 °C for 30 min. The supernatant was dried with nitrogen gas. The Residues were dissolved in 150 µL of 50% acetonitrile. The chromatographic separation was achieved by using the Atlantis Premier BEH Z‐HILIC column (100 × 2.1 mm, particle size 1.7 µm, Waters Corp.) at 30 °C with eluent A (water with 15 mм ammonium bicarbonate) and eluent B (90% acetonitrile with 15 mм ammonium bicarbonate) at 0.5 mL min^−1^ flow rate. Mass spectrometry analysis was performed by using the Waters SYNAPT XS in positive and negative ion ESI modes. The capillary voltage in positive ion mode was set at 2.5 kV, and that for negative ion mode was 2.0 kV. The desolvation gas flow rate was set at 800 L h^−1^. The desolvation and source temperatures were set at 500 °C and 120 °C, respectively.

### Mitochondrial Stress Analysis

Mitochondrial function was assessed using a Seahorse XFe Analyzer (Agilent, Santa Clara, CA, USA) and the XF Cell Mito Stress Test Kit (Agilent, 103015–100) following the manufacturer's instructions. Briefly, SAS (8000 cells/well) and MDA‐MB‐231 (20000 cells/well) cells were seeded to XF24 cell culture microplates (Agilent, 102340‐100). After the indicated treatment, the cell culture medium was exchanged with the Seahorse XF DMEM assay medium (Agilent, 103680‐100) supplemented with 25 mм glucose, 4 mм glutamine, and 2% FBS, and placed in a 37 °C incubator containing 100% atmosphere for 1 h. After calibration of the sensor cartridge, cell plates were loaded into the Seahorse XFe Analyzer, and oligomycin (1 µм), FCCP (1 µм for SAS and 2 µм for MDA‐MB‐231 cells), and Rot/AA (0.5 µм) were added to each well sequentially. The OCR was measured over a 100‐min time frame at 37 °C. Data analysis was done using Wave 2.6.3 software.

### Subcellular Fractionation

Cells on tissue culture plates were PBS‐washed and harvested by using cell scrapers. The cell pellet was then resuspended in a hypotonic buffer (20 mм Tris, pH 7.4, 10 mм NaCl, 3 mм MgCl_2_) and incubated on ice for 15 min. Triton X‐100 was then added to a 0.5% final concentration, and the samples were mixed using a vortex mixer at the highest setting for 10 s. After centrifugation (3000 rpm, 4 °C, 10 min), the supernatant was transferred to a new microtube as the cytosolic fraction. The pellet was resuspended in the lysis buffer (50 mм Tris, pH 7.4, 150 mм NaCl, 0.5% NP‐40) and then homogenized using the Qsonica S‐4000 Ultrasonic Sonicator (Misonix) equipped with a cup horn. After centrifugation (13000 rpm, 4 °C, 10 min), the supernatant was transferred to a new microtube as the nuclear fraction. Appropriate amounts of the cytosolic and the nuclear fractions were mixed with SDS loading dye and heated to 100 °C for 10 min. The samples were then subjected to SDS‐PAGE and Western blotting analysis.

### Transcriptomic Analysis

MDA‐MB‐231 cells were treated with the effectors for 24 h and harvested with TRIzol (Thermo Fisher Scientific, 15596018). After total RNA extraction, the transcriptomic analysis was done using the RNA Sequencing service of Taiwan Genomic Industry Alliance Inc. (TGIA).

### Soft Agar Colony Formation Assay

The bottom layer medium (2 mL/well DMEM medium with 0.7% melted agarose) was first plated into 6‐well plates. After solidifying the bottom layer, the top layer medium (1.5 mL/well DMEM medium with 0.35% melted agarose and cells) was plated on top of the bottom layer. The final cell density was 20000 cells/well for SAS and 50000 cells/well for MDA‐MB‐231 cells. After solidification, 1 mL of DMEM medium was added to each well to cover the top layer. Cells were incubated in a humidified CO_2_ incubator at 37 °C for 1 to 3 weeks to allow initial colony formation. The effectors were treated by exchanging the cover medium three times a week. After 3 to 4 weeks of effector treatment, the diameters of the colonies were measured.

### Statistical Analysis

Figure preparation and statistical calculation were done using GraphPad PRISM 7. Representative results and images from each experiment are presented. Data are shown as mean ± SD unless otherwise indicated. Statistical significance was calculated using two‐tailed unpaired Student's *t*‐tests (between two groups) or one‐way ANOVA with Tukey's multiple comparison tests (between more than three groups) unless otherwise stated.

## Conflict of Interest

The authors declare no conflict of interest.

## Author Contributions

W.C.W., H.J.K., M.H.Y., M.L.C., C.Y.L., Y.H., T.H., and T.J.C. conceived and designed the study. M.L.C. and C.J.L. conducted the metabolomic analysis and commented on the results. C.Y.L. and H.Y.H. performed the WGCNA analysis and commented on the data. T.J.C., M.J.W., W.Y.S., Y.C.H., C.H.L., Y.L.C., W.K.F., S.M.Y., and P.L.C. performed the experiments, analyzed the data, and interpreted the results. W.C.W. and T.J.C. wrote the manuscript.

## Supporting information



Supporting Information

## Data Availability

The data that support the findings of this study are available from the corresponding author upon reasonable request.
