# Supplementary material for: GSH as an A‐A Type Allosteric Activator of PKM2: Modulating Cancer Cell Homeostasis and Ferroptosis Susceptibility
Source: Adv Sci (Weinh). 2025 Nov 10;13(5):e19368. doi: 10.1002/advs.202519368 (PMC12850047; doi:10.1002/advs.202519368)
Supplement: Supplementary file 1 — Supporting Information [file ADVS-13-e19368-s001.docx]

Supporting Information

GSH as an A-A type allosteric activator of PKM2: modulating cancer cell homeostasis and ferroptosis susceptibility

Tsan-Jan Chen, Chi-Jen Lo, Meng-Jen Wu, Wei Yang Sit, Hsin-Yu Hsu, Yi-Cheng Huang, Chien-Hung Lu, Yu-Lun Chen, Wei-Kai Fang, Shan-Min Yang, Pei-Lien Chen, Tokuko Haraguchi, Yasushi Hiraoka, Chun-Yu Lin, Mei-Ling Cheng, Muh-Hwa Yang, Hsing-Jien Kung, and Wen-Ching Wang*


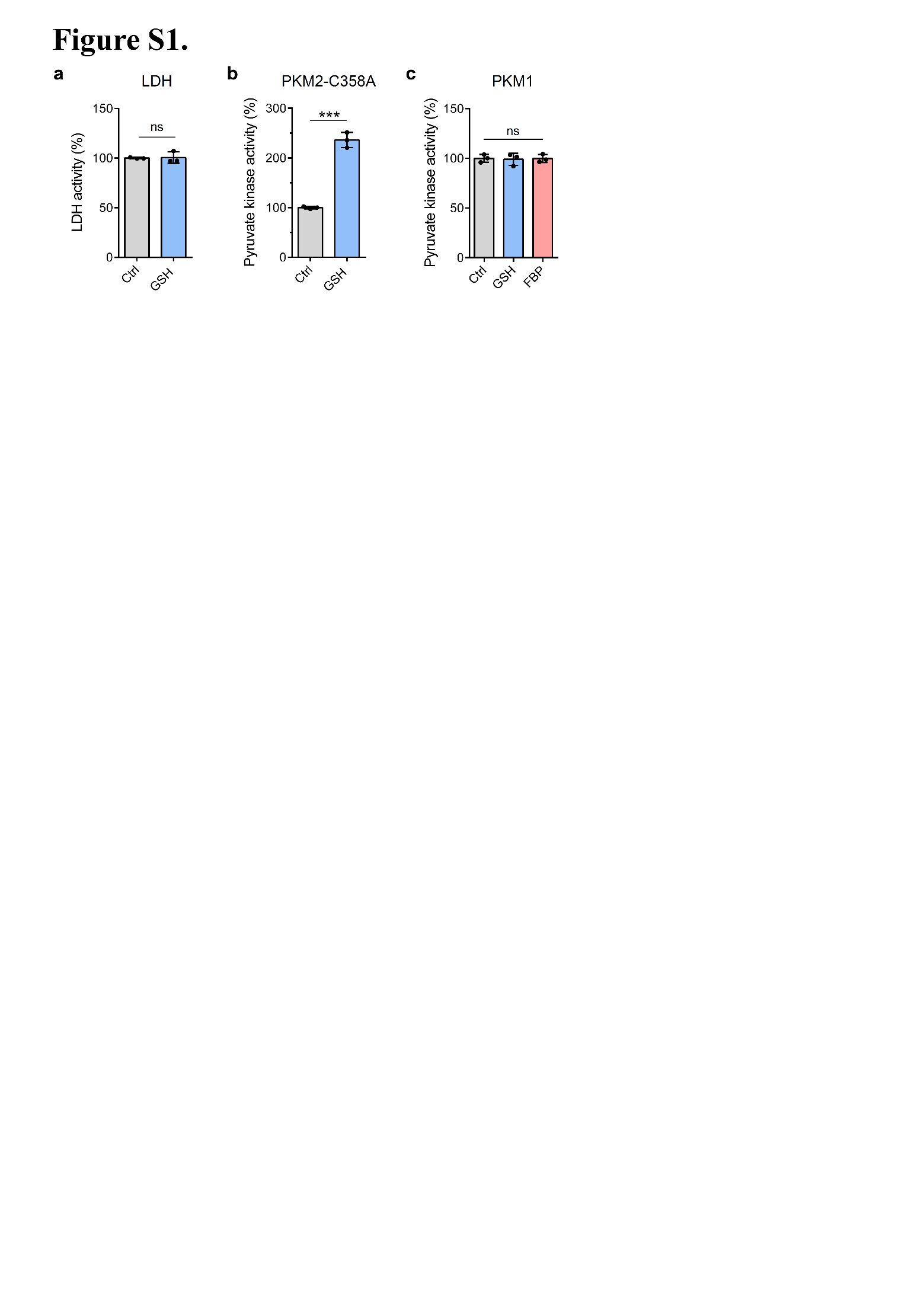


**Figure S1.** Effects of GSH on PKM2 enzymatic activity. a) LDH counter assay measured with or without 0.5 mм GSH (*n* = 3). b) Pyruvate kinase activity assay of PKM2-C358A mutant measured with or without 100 μм GSH (*n* = 3). c) Pyruvate kinase activity assay of PKM1 tested with GSH or FBP at 100 μм (*n* = 3). Data are shown as mean $\pm$ SD for (a)‒(c). Two-tailed unpaired Student’s *t*-test for (a) and (b). One-way ANOVA with Tukey’s multiple comparison test for (c). ****p* < 0.001, ns: *p* > 0.05.


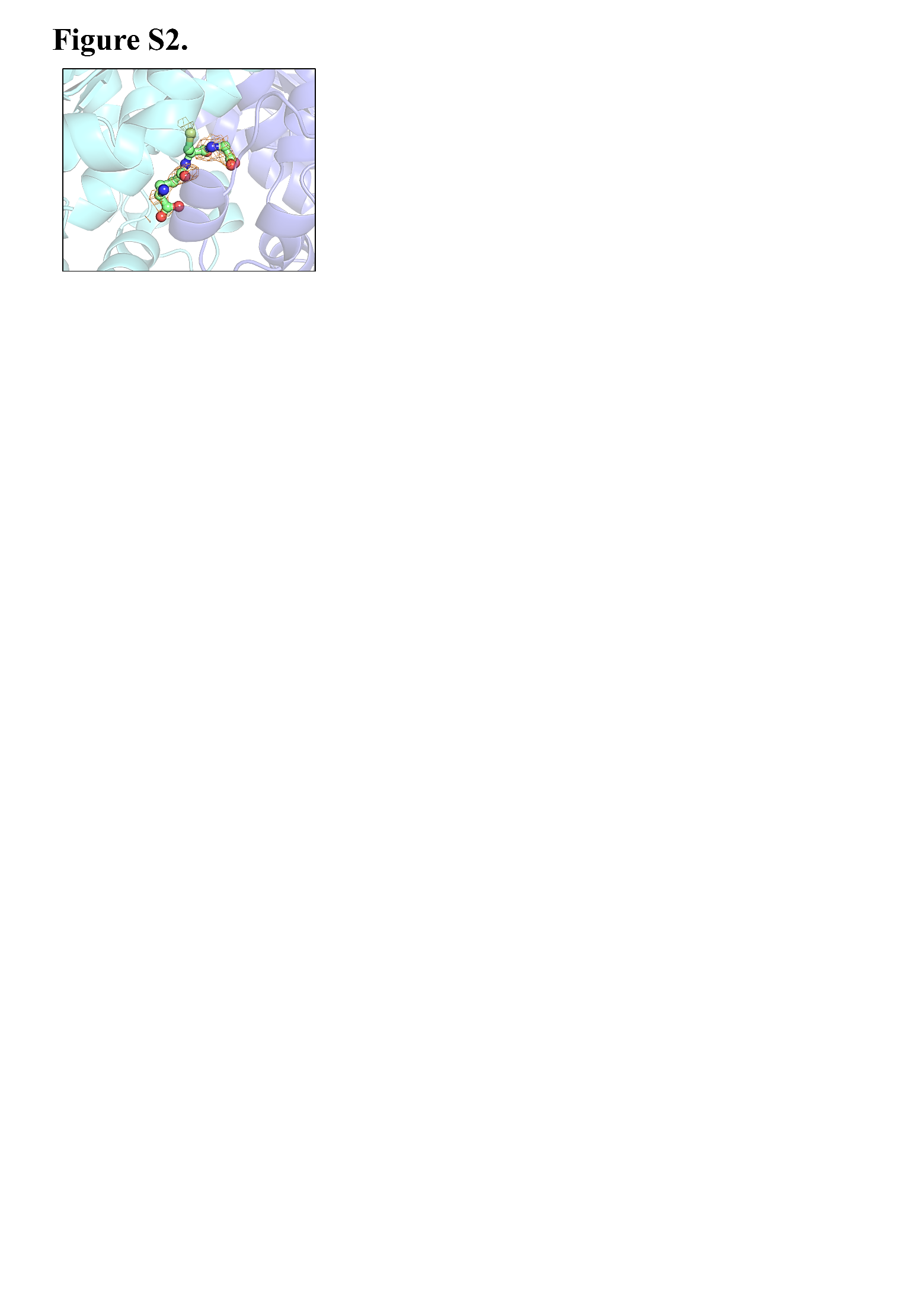


**Figure S2.** The omit map of GSH in the PKM2-GSH complex. The omit map of GSH, contoured at 1.1 σ, is shown as an orange mesh. This map was generated using Sfcheck 7.05.04 within the CCP4i package.


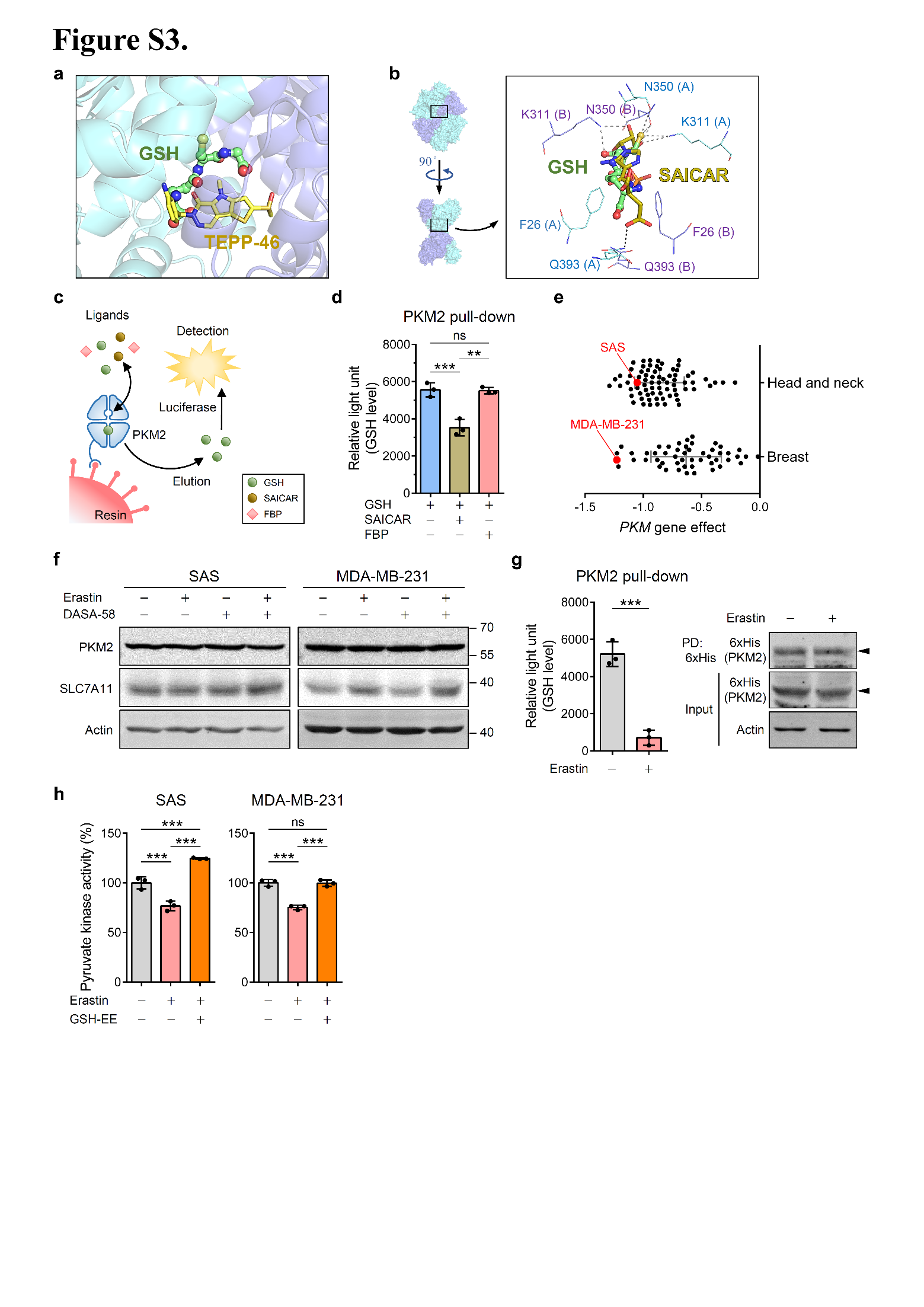


**Figure S3.** GSH, SAICAR, and pharmacological activators share the PKM2 A-A binding pocket, and erastin disrupts cellular GSH-PKM2 binding. a) Structural superimposition of GSH (shown as green stick-and-ball model in this study) and TEPP-46 (shown as yellow sticks; PDB: 3U2Z) within the PKM2 allosteric activator binding pocket, illustrating the overlapping binding sites of these two activators. b) Superimposition of GSH (green sticks and balls; this study) and docked SAICAR (ochre sticks) in the A-A interface pocket of PKM2. Residues from chains A and B are shown as thin lines (labeled in parentheses). Hydrogen bonds between each ligand and N350/K311 are indicated by gray dashed lines. The SAICAR-Q393 (A) hydrogen bond is indicated with a black dashed line. c‒d) *In vitro* GSH displacement assay. A schematic diagram showing the pull-down assay procedure (c). His-tagged PKM2 was pre-loaded with GSH, washed to remove unbound ligand, then incubated with 5 mм SAICAR or 5 mм FBP for 30 minutes. PKM2-bound GSH was quantified by luminescence assay and normalized to the no-competitor control (*n* = 3) (d). e) The *PKM* gene effects of head and neck cancer (*n* = 74) and breast cancer (*n* = 51) cell lines from DepMap portal are plotted. Lower gene effect indicates higher dependency on *PKM* gene. SAS and MDA-MB-231 cells are highlighted. f) Western blotting analysis of PKM2 and SLC7A11 protein levels in SAS and MDA-MB-231 cells treated with erastin (2 μм for SAS, 5 μм for MDA-MB-231) and/or DASA-58 (5 μм for SAS, 10 μм for MDA-MB-231) for 24 hours. g) Cell-based PKM2-bound GSH pull-down assay. HEK293T cells were transiently transfected with His-tagged PKM2, then treated with or without 10 μм erastin for 24 hours. His-tagged PKM2 was captured on cobalt-coated resin under native conditions, washed, and eluted. Co-purified GSH was quantified by luminescence assay. GSH levels were normalized to total PKM2 recovered. Untreated His-tagged PKM2 pull-downs contain high GSH, whereas erastin treatment significantly reduces both intracellular GSH and the amount recovered with PKM2 (*n* = 3). Immunoblotting analysis of input lysates and resin eluates probed for His tag (PKM2) and loading control (actin) is shown in the right panel. PD, pull-down. h) Rescue of PKM2 activity by GSH-EE replenishment. Pyruvate kinase activity was measured in SAS and MDA-MB-231 cell lysates and normalized to the PKM2 protein levels. Cells were pre-treated with erastin (5 μм for SAS; 10 μм for MDA-MB-231) for 24 hours, then incubated with 5 mм GSH-EE for 1 hour, followed by pyruvate kinase activity assays (*n* = 3). Data are shown as mean $\pm$ SD for (d), (g), (h). One-way ANOVA with Tukey’s multiple comparison tests for (d) and (h). Two-tailed unpaired Student’s *t*-test for (g). ***p* < 0.01, ****p* < 0.001, ns: *p* > 0.05.


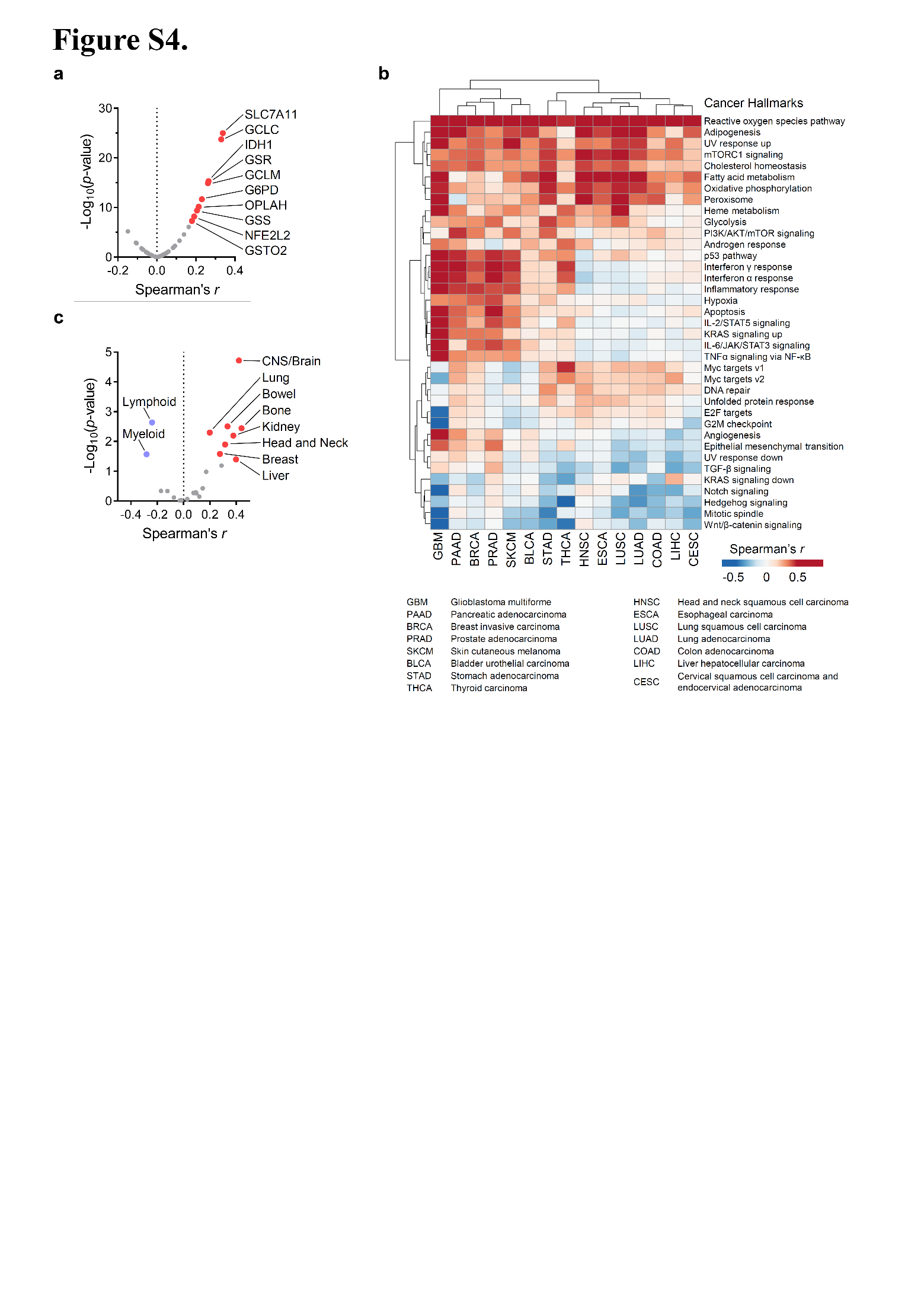


**Figure S4.** *PKM* and *SLC7A11* correlation analysis in TCGA and DepMap databases. a) The correlation between gene expression levels [Log_2_(TPM + 1)] and GSH abundance (Log_10_ scale) in 911 cell lines from the DepMap portal. The GLUTATHIONE_METABOLIC_PROCESS gene set (MSigDB) is used for the analysis. The Spearman’s correlation coefficient (*r*) and the -Log_10_(*p*-value) are shown. The top 10 positively correlated genes are indicated by red dots. b) Pan-cancer correlation analysis. The Spearman’s correlation coefficient (*r*) between each cancer hallmark signature and ferroptosis signature (MSigDB) in 15 cancer types (TCGA Pan-Cancer) is depicted as red (positive correlation) or blue (negative correlation) bars. The ferroptosis scores and each cancer hallmark score were calculated using the ssGSEA module of GenePattern. Hierarchical clustering was applied to the rows and columns. The abbreviations of TCGA studies are listed. c) Correlation between *PKM* and *SLC7A11* mRNA levels in different cancer cell lineages (DepMap portal). Positive and negative correlations with *p*-values below 0.05 are colored in red and blue, respectively. CNS, central nervous system.


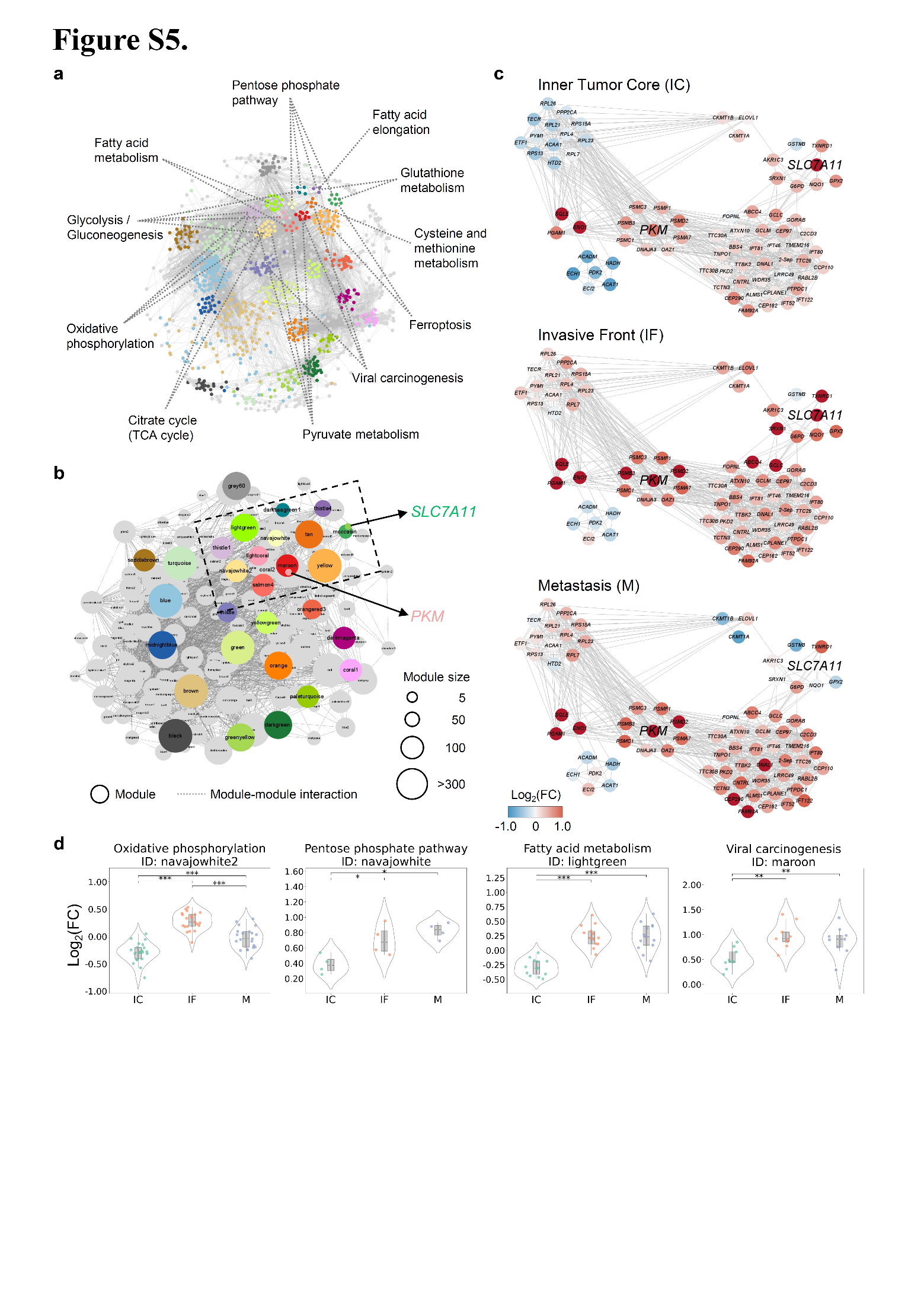


**Figure S5.** WGCNA and MMI network analysis. a) WGCNA gene correlation network from 65 HNSCC samples, consisting of 1697 nodes and 17986 edges. Nodes are color-coded by module membership. b) The MMI network reconstructed from the gene correlation network. The MMI network, comprising 168 modules (circles) and 2279 MMIs (dotted lines), was determined by the enrichment of inter-module co-expressed gene pairs based on the hypergeometric distribution. Module IDs are shown in the center of nodes. The size of the nodes is proportional to the module size (i.e., the number of genes). The MMI subnetwork centered on the modules of interest (IDs: maroon and moccasin), genes *PKM* and *SLC7A11* included, and their neighboring modules are highlighted by a dotted square. c) Subnetworks centered on *PKM* and *SLC7A11*, and the genes involved in the enriched modules are shown. The color key indicates Log_2_(tumor vs. normal fold change) [Log_2_(FC)] for IC, IF, and M in HNSCC samples. d) Violin plots showing Log_2_(FC) values for genes within modules enriched in pathways, including oxidative phosphorylation (ID: navajowhite2), pentose phosphate pathway (ID: navajowhite), fatty acid metabolism (ID: lightgreen), and viral carcinogenesis (ID: maroon). Each dot represents a gene’s Log_2_(FC) value within the module for the specified tumor site. The boxplot within each violin plot indicates the minimum, first quartile, median, third quartile, and maximum values. Statistical significance was assessed using the Wilcoxon signed-rank test. **p* < 0.05, ***p* < 0.01, ****p* < 0.001.


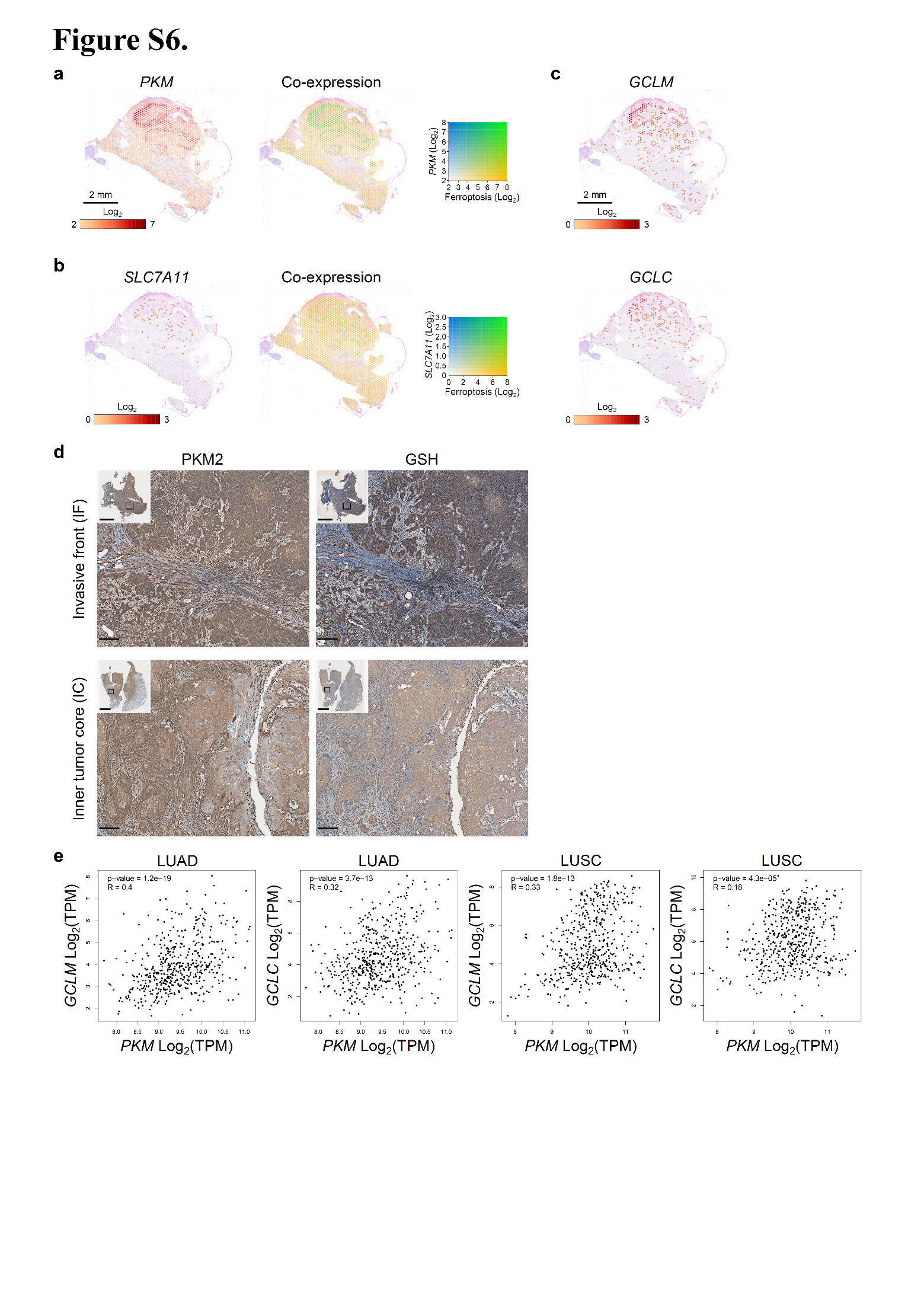


**Figure S6.** The spatial transcriptomic analysis of *PKM,* *SLC7A11*, and GSH-biosynthetic enzymes expression. a‒b) Spatial transcriptomic analysis using the GEO dataset: GSE181300. Heat maps illustrating the expression levels of *PKM* (a) and *SLC7A11* (b) across different tumor sites (left panel). The right panel displays the co-expression profiles of the ferroptosis pathway with *PKM* or *SLC7A11*, visualized as spatial co-expression maps in the HNSCC tumor section. c) Spatial transcriptomic maps of the same HNSCC section as (a). Heat maps illustrating the expression levels of the GSH-biosynthetic enzymes *GCLM* (top) and *GCLC* (bottom); red indicates high expression (Log_2_ scale). d) Representative IHC staining of PKM2 (left two panels) and GSH (right two panels) in HNSCC patient specimens. Upper row: invasive front; lower row: inner core. Brown signal = PKM2, GSH; blue = Mayer's hematoxylin counterstain. Scale bar = 5 mm for the full images at the upper left corners in each panel, and 200 µm for the enlarged images. e) Scatter plots of *PKM* versus *GCLM* or *GCLC* mRNA levels in TCGA cohorts (LUAD and LUSC). Each point represents one tumor sample. Spearman’s correlation coefficient (*r*) and *p*-values are shown. The figures were generated by the GEPIA2 server.


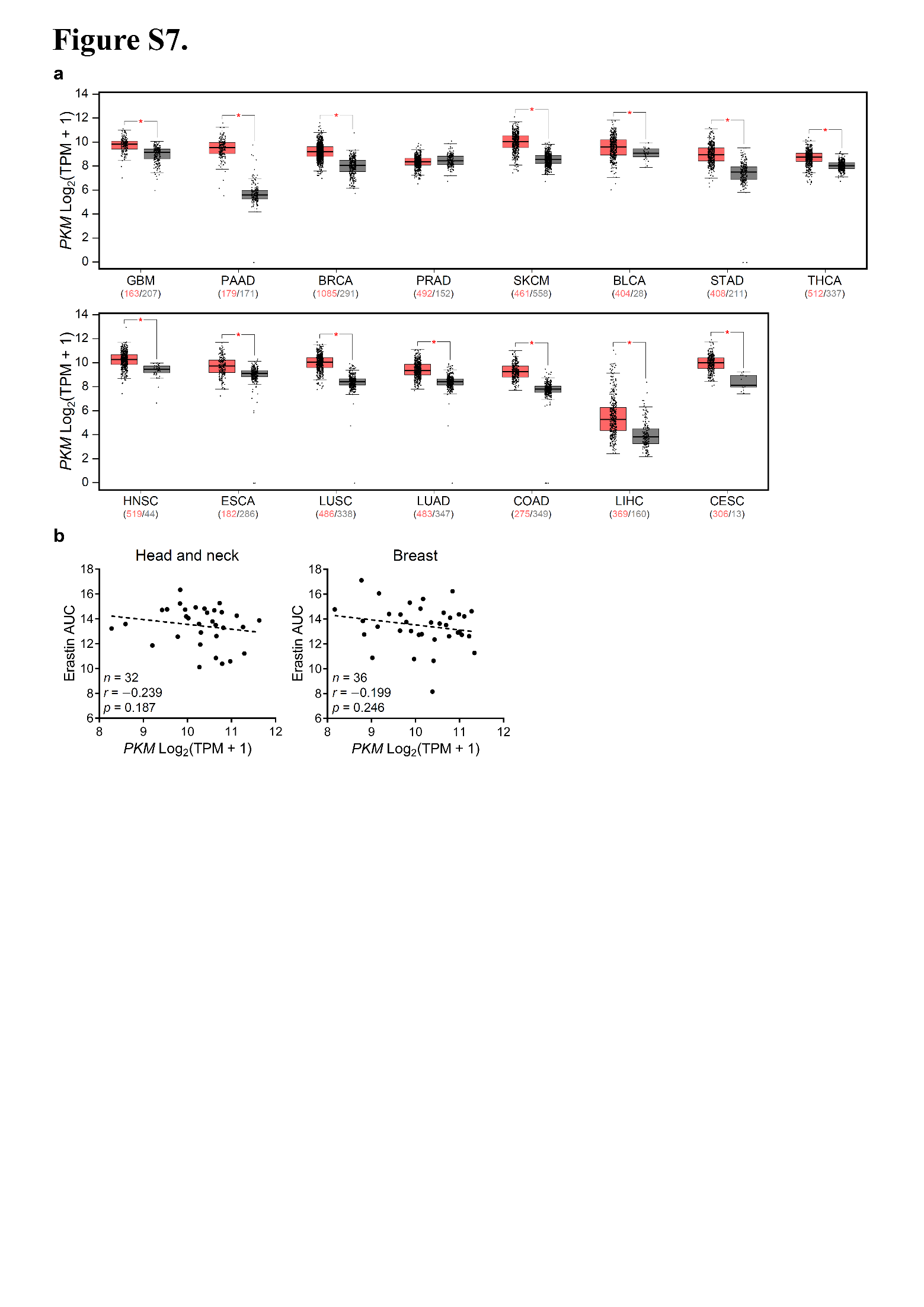


**Figure S7.** *PKM* expression across various cancers and its relationship to erastin sensitivity. a) Boxplots of *PKM* mRNA levels [Log₂(TPM + 1)] in tumor (red) versus matched normal (gray) tissues for 15 TCGA cohorts. Cancer abbreviations with *n*(Tumor)/*n*(Normal) are indicated below each plot. The figures were generated by the GEPIA2 server. **p* < 0.05. b) Scatter plots of *PKM* mRNA expression [Log₂(TPM + 1)] versus erastin sensitivity (area under the dose-response curve, AUC) in head and neck cancer cell lines (left; *n* = 32) and breast cancer cell lines (right; *n* = 36). Spearman’s correlation coefficient (*r*) and *p*-values are indicated on each plot (DepMap portal).


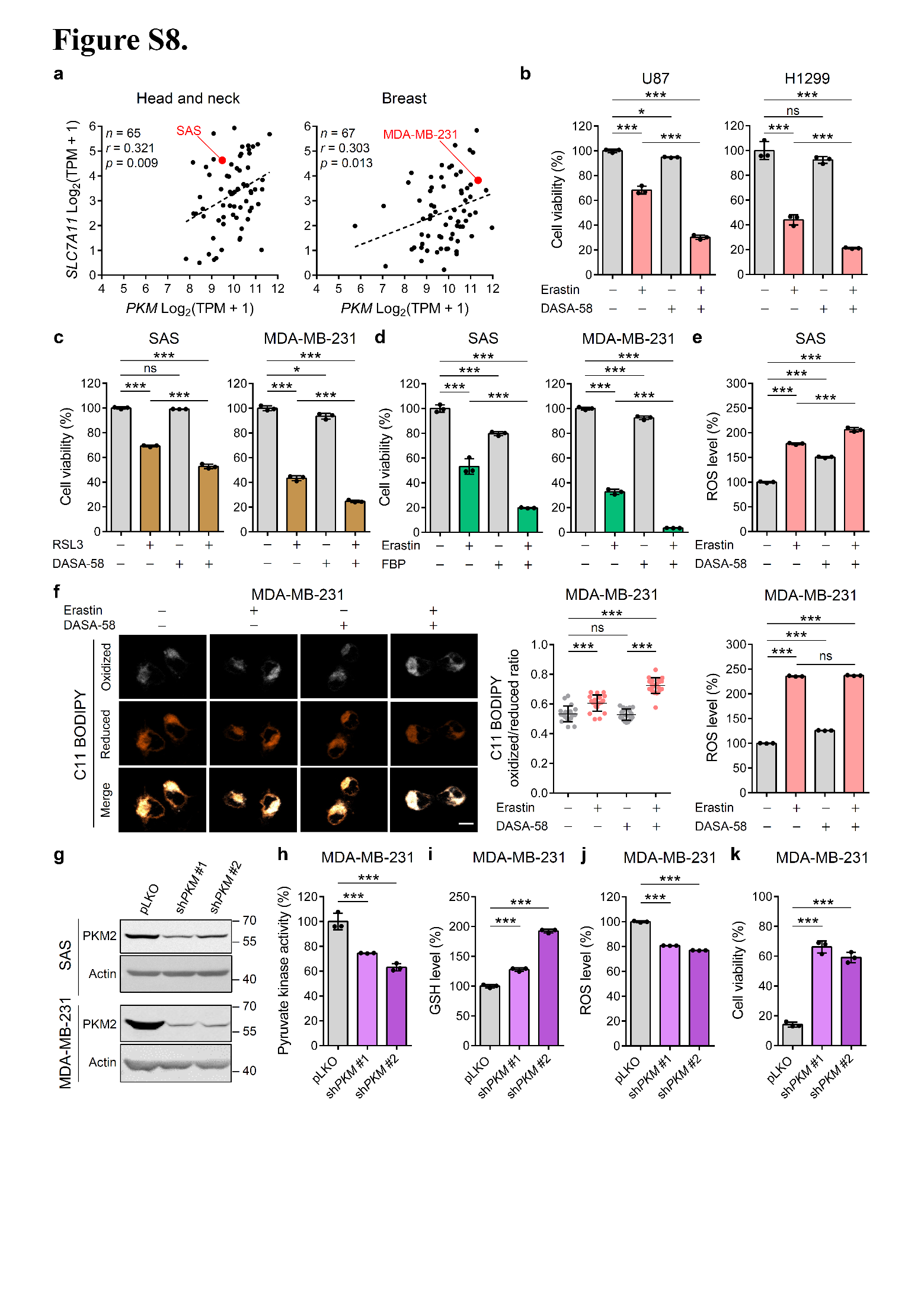


**Figure S8.** Co-targeting PKM2 activation and ferroptosis in various cancer cell lines. a) Correlation analysis of *PKM* and *SLC7A11* mRNA expression in breast and head and neck cancer cell lines. Expression data was retrieved from the DepMap portal. The Spearman’s correlation coefficient (*r*) and the *p*-values are shown. The dashed line represents the linear regression trendline, showing the overall correlation between *PKM* and *SLC7A11* expression in these cell lines. b) Cell viability of U87 glioblastoma and H1299 non-small cell lung carcinoma cells following 24 hours of treatment with erastin (2.5 μм for U87; 2 μм for H1299) with or without 10 μм DASA-58, measured by MTT assay (*n* = 3). c) Cell viability of SAS and MDA-MB-231 cells treated with RSL3 (3 μм for SAS; 0.5 μм for MDA-MB-231) with or without 20 μм DASA-58 for 24 hours (*n* = 3). d) Cell viability of SAS and MDA-MB-231 cells following treatment with erastin (4 μм for SAS and MDA-MB-231) with or without 5 mм FBP for 24 hours (*n* = 3). e) Measurement of ROS levels by H2DCFDA staining. Cells were treated with combinations of erastin and DASA-58 for 24 hours (SAS: 2 μм erastin/5 μм DASA-58; MDA-MB-231: 5 μм erastin/10 μм DASA-58; *n* = 3). f) Fluorescent imaging of C11 BODIPY staining to assess lipid peroxidation. MDA-MB-231 cells were treated with 5 μм erastin and/or 5 μм DASA-58 for 24 hours. Representative images show oxidized (white) and reduced (red) C11 BODIPY signals (left panel). Scale bar = 10 μm. The oxidized to reduced C11 BODIPY fluorescence intensity ratio is quantified (right panel, *n* = 20). g‒k) Effects of PKM2 knockdown in SAS and MDA-MB-231 cells. Western blotting analysis of pLKO and sh*PKM* cells (g). Pyruvate kinase activity of MDA-MB-231 pLKO and sh*PKM* cells normalized to total protein level (*n* = 3) (h). Intracellular GSH levels of MDA-MB-231 pLKO and sh*PKM* cells normalized to total protein level (*n* = 3) (i). ROS levels of MDA-MB-231 pLKO and sh*PKM* cells normalized to total protein level (*n* = 3) (j). Cell viability in MDA-MB-231 pLKO and sh*PKM* cells treated with 10 μм erastin for 24 hours, assessed by MTT assay (*n* = 3). Untreated groups (data not shown) are defined as 100 % cell viability (k). Data are shown as mean $\pm$ SD for (b)‒(f) and (h)‒(k). One-way ANOVA with Tukey’s multiple comparison test for (b)‒(f) and (h)‒(k). **p* < 0.05, ***p* < 0.01, ****p* < 0.001, ns: *p* > 0.05.


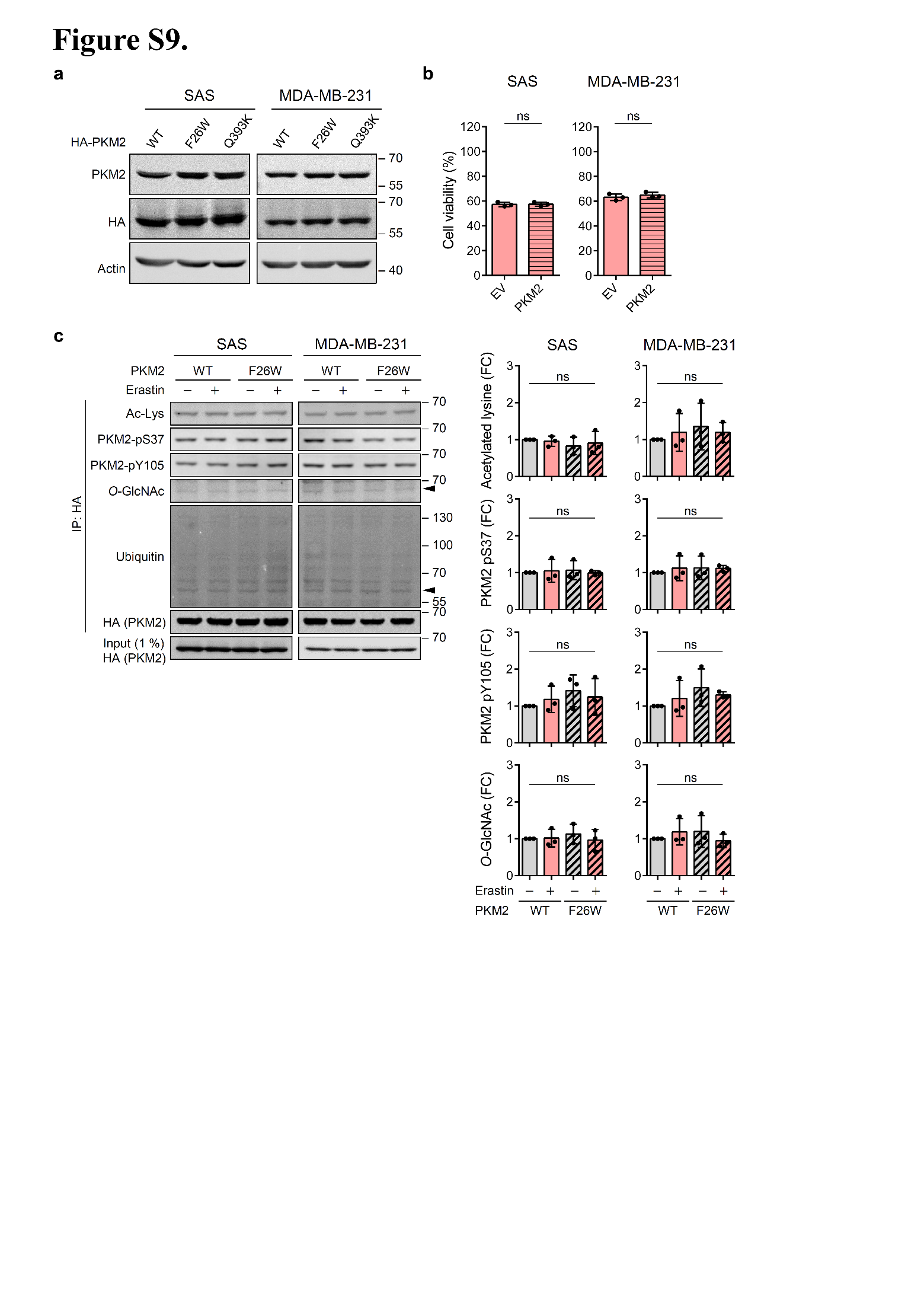


**Figure S9.** Functional analysis and PTM profiling of PKM2 GSH-binding mutants. a) Western blots of PKM2-knockdown SAS and MDA-MB-231 cells re-expressing HA-tagged PKM2 WT, F26W, or Q393K. Top: anti-PKM2; middle: anti-HA; bottom: actin loading control. b) Viability of parental cells transfected with empty vector (EV) versus HA-PKM2, treated with erastin (15 μм for SAS and MDA-MB-231) for 24 hours (*n* = 3). c) PKM2 PTM profiling. After 12 hours of treatment (with or without erastin), HA-tagged WT or F26W PKM2 were immunoprecipitated, and the PTM levels (acetylated lysine, pS37, pY105, *O*-GlcNAcylation, and ubiquitination) were analyzed by Western blotting. Representative blots are shown in the left panel, and quantification of PTM levels (fold change relative to untreated WT) is presented in the right panel (*n* = 3). Ubiquitination was not quantified due to weak signals. Ac-Lys, acetylated lysine. Data are shown as mean $\pm$ SD for (b) and (c). Two-tailed unpaired Student’s *t*-test for (b). One-way ANOVA with Tukey’s multiple comparison test for (c). ns: *p* > 0.05.


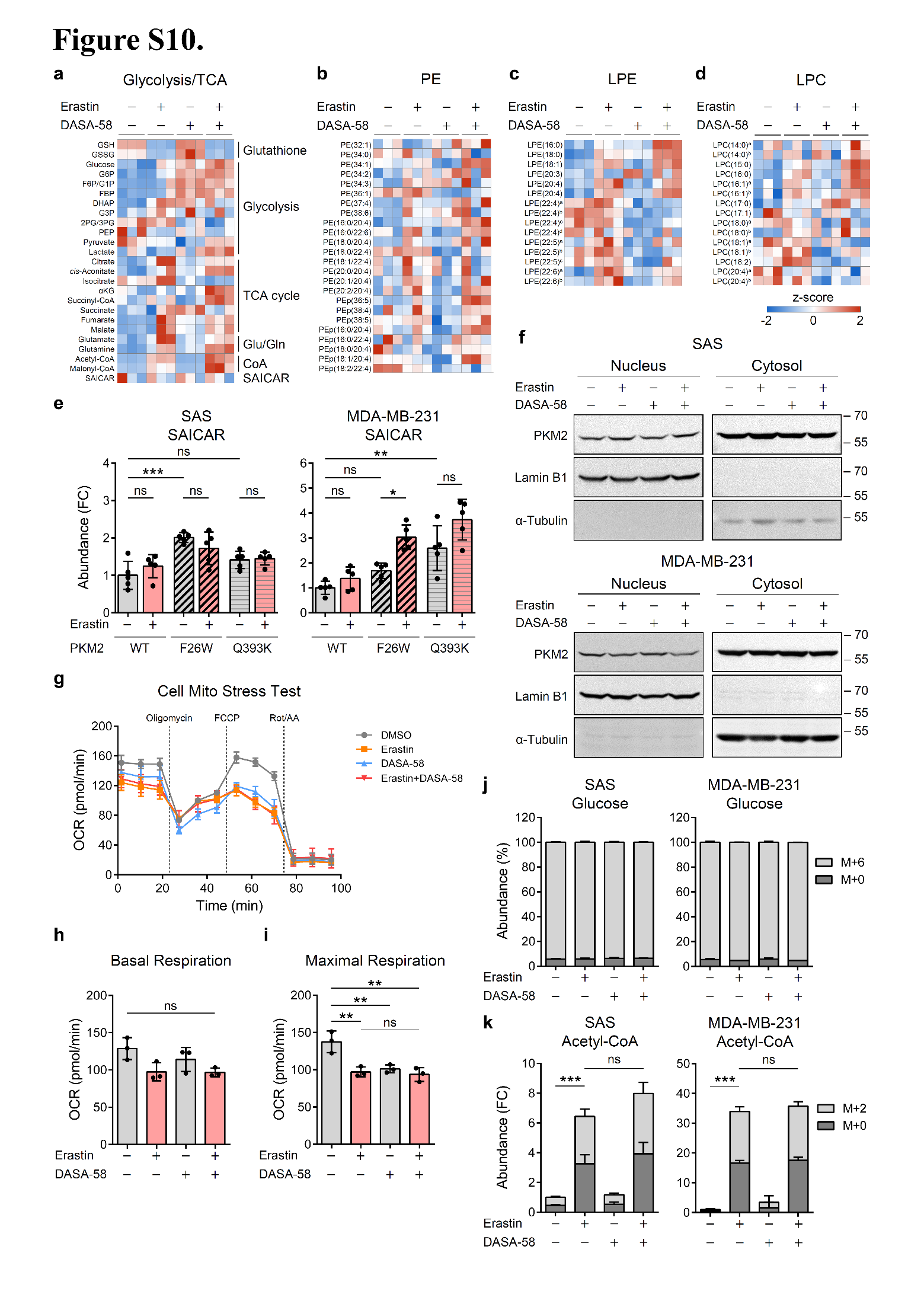


**Figure S10.** Metabolomic analyses reveal metabolic reprogramming induced by erastin and DASA-58. a‒d) Heatmaps showing the abundance of central carbon metabolites (a), PE species (b), LPE species (c), and LPC species (d). SAS cells were treated with the effector combinations (2 μм erastin/5 μм DASA-58) for 24 hours. Following treatment, cells were harvested for metabolomic analysis. The metabolite abundance was normalized to total protein levels (*n* = 3). The superscripts indicate isomers that are indistinguishable. e) Intracellular SAICAR levels in *PKM*-depleted SAS and MDA-MB-231 cells reconstituted with WT, F26W, or Q393K PKM2. Cells were treated with DMSO or erastin (5 μм for SAS and 10 μм for MDA-MB-231) for 24 hours, followed by mass spectrometry analysis (*n* = 5). f) Subcellular fractionation analysis of SAS and MDA-MB-231 cells treated with various combinations of erastin and/or DASA-58 (SAS: 2 μм erastin/5 μм DASA-58 for 24 hours; MDA-MB-231: 5 μм erastin/10 μм DASA-58 for 24 hours). Cells were fractionated into nuclear and cytoplasmic compartments, and protein extracts were analyzed by Western blotting to assess PKM2 localization and related protein distribution. g‒i) Mitochondrial respiration analysis in SAS using Seahorse XF Analyzer: OCR profiles after treatment (2 μм erastin/5 μм DASA-58) in the presence of oligomycin, FCCP, and Rot/AA (g); basal respiration (h); and maximal respiration (i). j) 13C_6_-Glucose labeling assay. After 24 hours of erastin and/or DASA-58 treatment, cells were labeled with 13C_6_-glucose for 30 minutes. The cells were harvested for metabolomic analysis. The ratio of labeled (M+6) and unlabeled (M+0) glucose is shown (*n* = 3). k) Following the same analysis in (j), the levels of labeled (M+2) and unlabeled (M+0) acetyl-CoA are shown as fold change (FC) (*n* = 3). The significance of M+2 species is indicated. Data are shown as mean $\pm$ SD for (e) and (g)‒(k). One-way ANOVA with Tukey’s multiple comparison test for (e), (h), (i), and (k). **p* < 0.05, ***p* < 0.01, ****p* < 0.001, ns: *p* > 0.05.


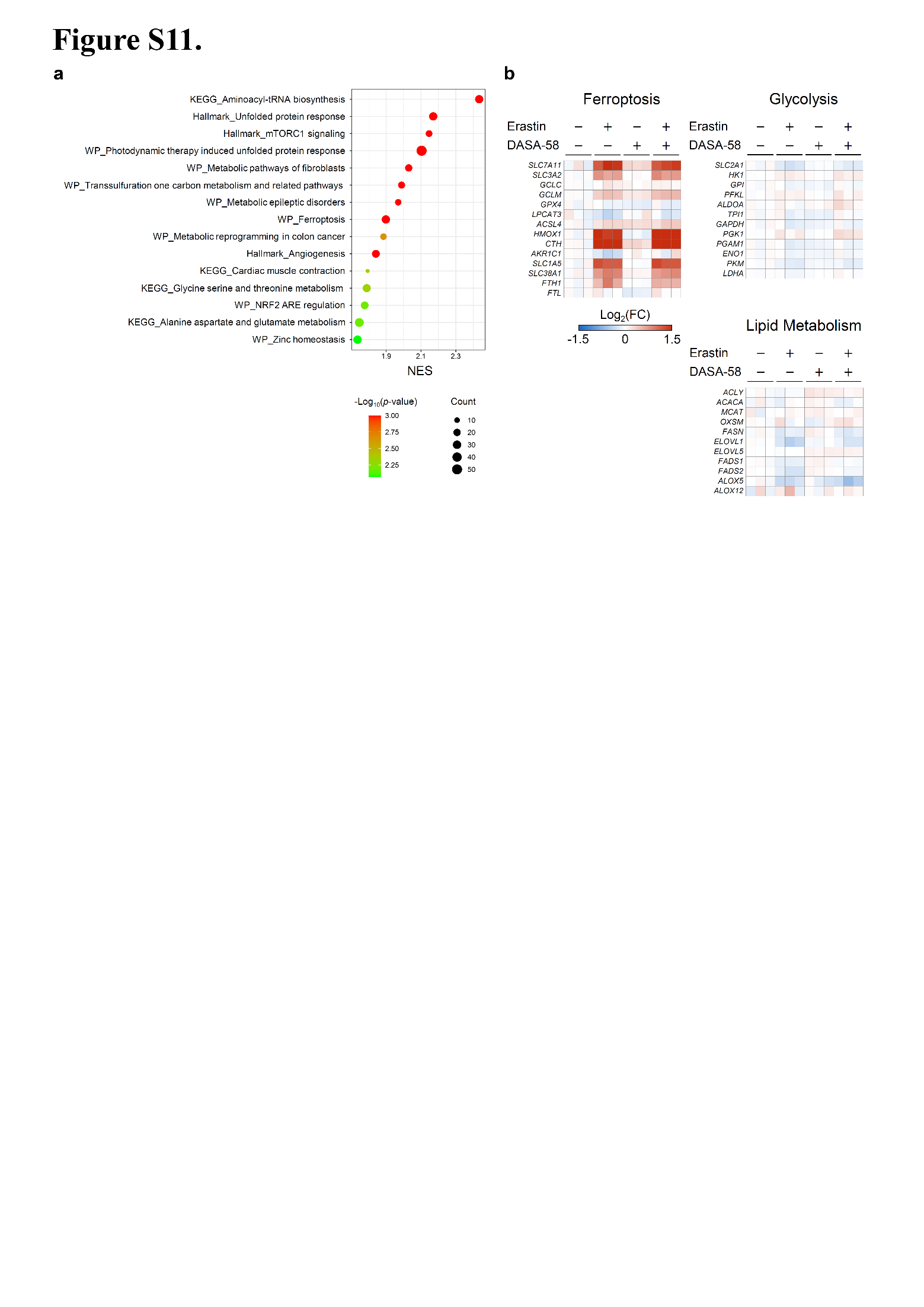


**Figure S11.** RNA-seq profiling under erastin and DASA-58 treatment. a) MDA-MB-231 cells were treated with erastin (10 μм) and/or DASA-58 (20 μм) for 24 hours, followed by total RNA extraction and RNA-seq analysis. Gene set enrichment analysis (GSEA) of RNA-seq data (*n* = 3) comparing the erastin/DASA-58 co-treatment group to the control. The analysis was performed using Hallmark, KEGG, and Wikipathway (WP) gene sets from MSigDB. The size of each data point represents the number of genes contributing to enrichment, while the color indicates the normalized enrichment score (NES). b) Heatmap displaying gene expression changes in response to erastin/DASA-58 co-treatment. The color key represents the Log₂ fold-change (FC) relative to the mean expression level of the untreated group.


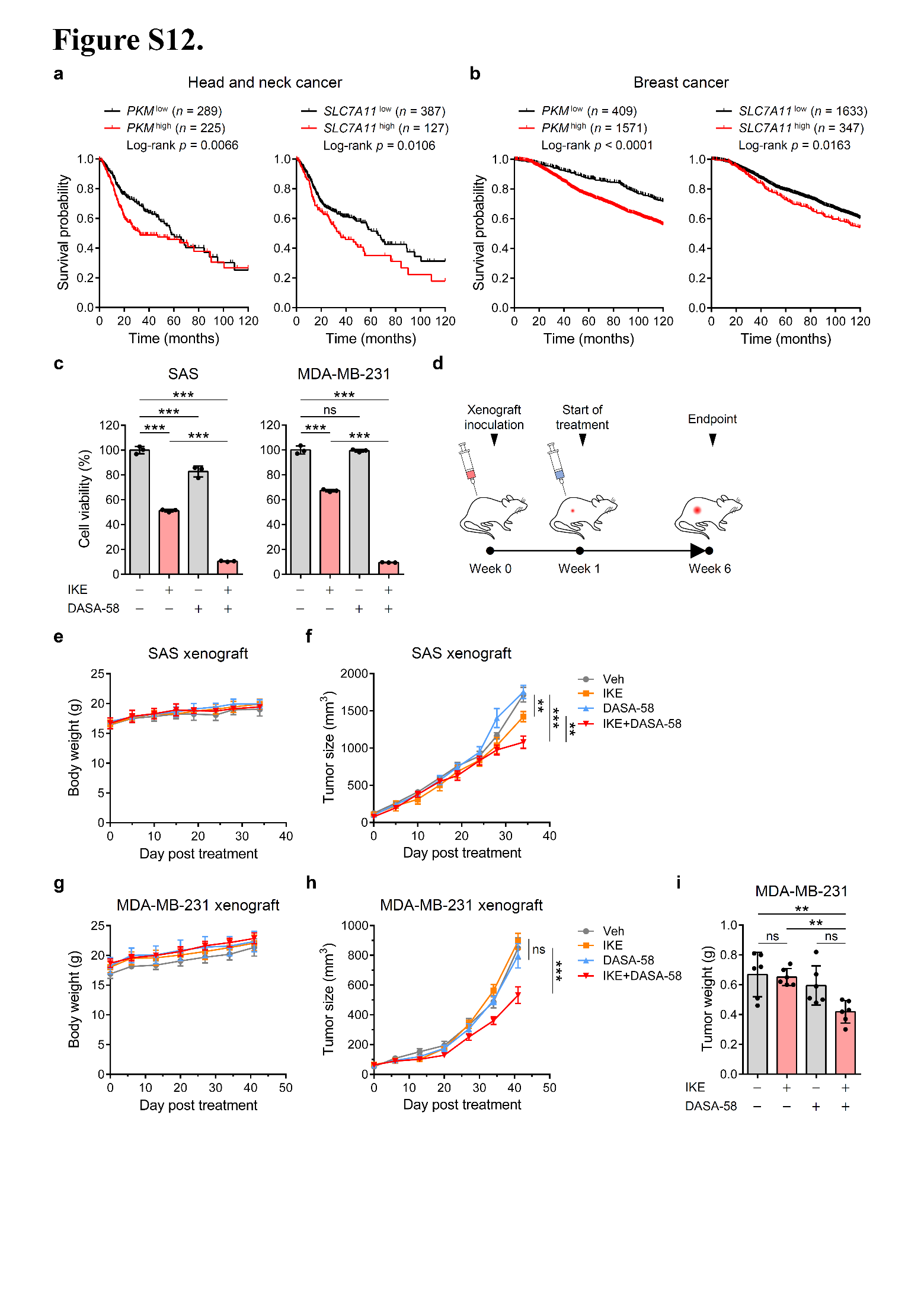


**Figure S12.** Combined targeting of PKM2 and SLC7A11 enhances ferroptosis sensitivity and reduces tumor growth *in vivo*. a‒b) Overall survival rate analysis of a head and neck cancer cohort (TCGA Pan-Cancer) and a breast cancer cohort (METABRIC) based on *PKM* and *SLC7A11*. Optimal cutoff values for *PKM* and *SLC7A11* were determined using Youden’s index. c) Cell viability measured by MTT assay in SAS and MDA-MB-231 cells treated with various combinations of effectors for 24 hours (2 μм IKE for SAS; 4 μм IKE for MDA-MB-231; 20 μм DASA-58) (*n* = 3). d) Schematic diagram of the experimental design of the xenograft mouse model. SAS tumors were treated with vehicle, IKE, DASA-58, or a combination of IKE and DASA-58 for approximately five weeks. e‒f) SAS xenograft study: body weight over time (*n* = 4) (e) and tumor volume growth curves (*n* = 4) (f). g‒i) MDA-MB-231 xenograft study: body weight over time (*n* = 3) (g), tumor volume growth curves (*n* = 6) (h), and tumor weights at endpoint (*n* = 6) (i). Data are shown as mean $\pm$ SD for (c), (e), (g), and (i), and mean $\pm$ SEM for (f) and (h). One-way ANOVA with Tukey’s multiple comparison test for (c) and **(**i). Two-way ANOVA with Tukey’s multiple comparison test for (f) and (h). ***p* < 0.01, ****p* < 0.001, ns: *p* > 0.05.

**Table S1.** Structural statistics for the PKM2-GSH complex.

| **Structure** |  |
| --- | --- |
| Structure name | PKM2-GSH |
| PDB code | 9IQQ |
| **Data collection** |  |
| Space group | P2_1_ |
| Unit Cell |  |
| *a*, *b*, *c* (Å) | 73.824, 130.288, 104.456 |
| *α*, *β*, *γ* (°) | 90.00, 93.97, 90.00 |
| Resolution (Å)^a)^ | 30.00-2.70 (2.80-2.70) |
| Unique reflections | 54936 |
| Completeness (%)^a)^ | 98.4 (99.4) |
| Average *I*/*σ* (I)^a)^ | 11.8 (2.0) |
| Redundancy^a)^ | 4.1 (4.2) |
| R_merge_(%)^a,b)^ | 11.0 (49.5) |
| **Refinement** |  |
| R value (%)^c)^ | 23.1 |
| R_free_ value (%)^d)^ | 28.2 |
| RMSD bond lengths (Å)^e)^ | 0.005 |
| RMSD bond angles (º)^e)^ | 1.364 |
| Ramachandran analysis (%)^f)^ |  |
| Preferred | 95.78 |
| Allowed | 3.87 |
| Outliers | 0.35 |
| ^a)^Values in parentheses refer to statistics in the highest-resolution shell; ^b)^R_merge_ = Σ\|I - <I>\|/ Σ(I); ^c)^R = Σ\|F_obs_-F_calc_\| ∕ ΣF_obs,_ where F_obs_ and F_calc_ are the observed and calculated structure-factor amplitudes, respectively; ^d)^R_free_ was computed using 5% of the data assigned randomly; ^e)^Root mean square deviation; ^f)^Estimated standard uncertainties based on maximum likelihood. | |

**Table S2.** Hydrogen bonding interactions between PKM2 and GSH.

| **PKM2 chain** | **PKM2**  **residue [atom]** | **GSH**  **residue [atom]** | **Hydrogen bond distance (Å)** |
| --- | --- | --- | --- |
| A | N350 [ O  ] | GSH  [ S^γ2^] | 3.51 |
|  | N350 [ O^δ1^] | GSH  [ S^γ2^] | 3.37 |
|  | D354 [ O^δ1^] | GSH  [ S^γ2^] | 3.14 |
|  | K311 [ N^ζ^ ] | GSH  [ S^γ2^] | 3.45 |
|  | L353 [ O  ] | GSH  [ N^1^ ] | 3.61 |
|  | L394 [ N  ] | GSH  [ O^12^] | 3.51 |
| B | N350 [ O^δ1^] | GSH  [ S^γ2^] | 3.83 |
|  | K311 [ N^ζ^ ] | GSH  [ O^2^ ] | 2.90 |
| PKM2-GSH hydrogen bonding interactions were analyzed by PDBePISA interface (v1.52). | | | |
